# Identification and Characterization of Key Differentially Expressed Genes Associated With Metronomic Dosing of Topotecan in Human Prostate Cancer

**DOI:** 10.3389/fphar.2021.736951

**Published:** 2021-12-06

**Authors:** Taraswi Mitra Ghosh, Jason White, Joshua Davis, Suman Mazumder, Teeratas Kansom, Elena Skarupa, Grafton S. Barnett, Gary A. Piazza, R. Curtis Bird, Amit K. Mitra, Clayton Yates, Brian S. Cummings, Robert D. Arnold

**Affiliations:** ^1^ Department of Drug Discovery and Development, Harrison School of Pharmacy, Auburn University, Auburn, AL, United States; ^2^ Department of Biology and Center for Cancer Research, Tuskegee University, Tuskegee, AL, United States; ^3^ Center for Pharmacogenomics and Single-Cell Omics, Auburn University, Auburn, AL, United States; ^4^ Department of Pathobiology, College of Veterinary Medicine, Auburn University, Auburn, AL, United States; ^5^ UAB O’Neal Comprehensive Cancer Center, University of Alabama at Birmingham School of Medicine, Birmingham, AL, United States; ^6^ Department of Pathology, University of Alabama at Birmingham School of Medicine, Birmingham, AL, United States; ^7^ Department of Pharmaceutical Sciences, Eugene Applebaum College of Pharmacy and Health Sciences, Wayne State University, Detroit, MI, United States

**Keywords:** topotecan, metronomic, prostate cancer, mCRPC, transcriptomics, RNA sequencing, TCGA

## Abstract

Repetitive, low-dose (metronomic; METRO) drug administration of some anticancer agents can overcome drug resistance and increase drug efficacy in many cancers, but the mechanisms are not understood fully. Previously, we showed that METRO dosing of topotecan (TOPO) is more effective than conventional (CONV) dosing in aggressive human prostate cancer (PCa) cell lines and in mouse tumor xenograft models. To gain mechanistic insights into METRO-TOPO activity, in this study we determined the effect of METRO- and CONV-TOPO treatment in a panel of human PCa cell lines representing castration-sensitive/resistant, androgen receptor (+/−), and those of different ethnicity on cell growth and gene expression. Differentially expressed genes (DEGs) were identified for METRO-TOPO therapy and compared to a PCa patient cohort and The Cancer Genome Atlas (TCGA) database. The top five DEGs were SERPINB5, CDKN1A, TNF, FOS, and ANGPT1. Ingenuity Pathway Analysis predicted several upstream regulators and identified top molecular networks associated with METRO dosing, including tumor suppression, anti-proliferation, angiogenesis, invasion, metastasis, and inflammation. Further, the top DEGs were associated with increase survival of PCa patients (TCGA database), as well as ethnic differences in gene expression patterns in patients and cell lines representing African Americans (AA) and European Americans (EA). Thus, we have identified candidate pharmacogenomic biomarkers and novel pathways associated with METRO-TOPO therapy that will serve as a foundation for further investigation and validation of METRO-TOPO as a novel treatment option for prostate cancers.

## Introduction

Prostate cancer (PCa) is the second leading cause of non-cutaneous cancer related deaths in men in the United States (www.cancer.org). PCa in individuals with precancerous, indolent, or slow growing malignant disease can evolve over many years. For those with advancing localized disease the standard treatment includes radical prostatectomy and radiation therapy with or without hormonal manipulation (Magnan et al., 2015; Hamdy et al., 2016). However, many patients ultimately develop resistance to hormone ablation therapy (androgen deprivation therapy; ADT) and are referred to as having: “non-metastatic castration-resistant prostate cancer” (nmCRPC) or metastatic CRPC (mCRPC) (Scher et al., 2015). The 2020 estimated transition of non-castrate state to mCRPC is less than 15%, with an estimated mortality rate of 19.5% (Scher et al., 2015). The use of enzalutamide, a 2^nd^ generation antiandrogen therapy, in men with castration-resistant prostate cancer after chemotherapy showed clinical activity, however, PSA levels increased in majority patients whose disease had progressed again (Scher et al., 2012). Further, patients receiving enzalutamide, apalutamide and abiraterone treatment will ultimately develop resistance (Galletti et al., 2017; Annala et al., 2018; Wang et al., 2021). While the *de novo* prevalence of neuroendocrine prostate cancer (NEPC) is limited (<2.0%), neuroendocrine differentiation (NED) following ADT to aggressive treatment-resistant NEPC forms of variant PCa have been estimated to be more than 25% (Yadav et al., 2017). Standard treatment for CRPC includes sipuleucel-T, abiraterone acetate plus prednisone (AA/P), or chemotherapy with docetaxel (Heidenreich et al., 2014; Cornford et al., 2017). Cabazitaxel, AA/P, enzalutamide, and radium-223 are available for second-line treatment of CRPC following docetaxel (Heidenreich et al., 2014; Cornford et al., 2017) and increases median overall survival (OS) by less than a year (De Bono et al., 2010; Oudard et al., 2017; van den Bergh et al., 2016).

African America (AA) men in general are more likely to progress or be diagnosed with mCRPC, fail to respond to conventional therapies, and show reduced survival compared with other ethnicities (seer.cancer.gov). Immunotherapy, has been shown to increase OS in patients with mCRPC by 4–5 months (Hall et al., 2011). Recent studies reported that AA patients responded to immunotherapy who received sipuleucel-T for mCRPC (Sartor et al., 2020) and abiraterone (Ramalingam et al., 2017), but overall treatment options remain limited and survival is poor. However, progression and PCa specific mortality was greater across Gleason scores (<6) in Black *vs* Nonblack men (Powell et al., 2010; Tsivian et al., 2013). These differences suggest that AA patients’ disease progression, sensitivity and response to chemotherapy are distinctive and novel approaches to identify effective treatment schedules are needed. This is largely due to the heterogeneity of individual tumors representing divergent genetic profiles and molecular signatures (Tannock, 2014). Furthermore, genomic complexity and the host/tumor microenvironment may contribute to treatment resistance (Hanahan and Weinberg, 2011). Therefore, it is necessary to develop new therapeutic approaches to overcome drug resistance, improve efficacy and increase overall survival in patients with nmCRPC, mCRPC, and NEPC.

Low-dose continuous drug exposure using METRO-like chemotherapy involves frequent administration of chemotherapeutic agents at low/fractionated doses at close intervals over prolonged periods of time (Kerbel and Shaked, 2017; Simsek et al., 2019). METRO is an emerging treatment option that has shown promise for various cancer types, including PCa (Fontana et al., 2009; Nelius et al., 2010). METRO has been reported to increase antitumor activity by inhibiting angiogenesis (decreasing tumor vascular density), increase tumor hypoxia or normalization of the tumor vasculature to improved blood flow and drug delivery (Aljuffali et al., 2011). Previously we demonstrated that METRO of topotecan (TOPO) *via* two different delivery schedules inhibited *in vivo* tumor growth using a xenograft model of human metastatic, NEPC in athymic NCR mice (Aljuffali et al., 2011). This was a serendipitous finding as we had chosen TOPO initially because it was not used clinically for PCa (Hudges, et al., 1995), and was not considered to have antiangiogenic activity at that time. Others had suggested that METRO therapy of drugs, including paclitaxel were able to promote the reduction of T-regulatory cells and reduce tumor growth in comparison to conventional schedules based on the maximum tolerated dose (Tanaka et al., 2009). Moreover, the antitumor activity observed in preclinical model lacking functional T-cells did not correlate with decrease tumor vascular density (Tanaka et al., 2009). While the mechanisms are not clear, the clinical benefits of METRO have been demonstrated with several other drugs in a variety cancers, including mCRPC, with varying degrees of success (Gebbia et al., 2011; Jellvert et al., 2011). Furthermore, low dose oral TOPO has been shown to be potent against several cancers, with its greatest efficacy for patients with ovarian cancer (Merritt et al., 2009; Wang et al., 2013; Jedeszko et al., 2015).

Alterations in gene expression levels have been reported to be associated with response to cancer drugs, including PCa. Recent studies identified angiogenesis genes CD31, VEGF, HIF-1*α*, and CEPs and apoptotic genes Bcl-2, Bax, and caspase-3 as associated with METRO treatment efficacy (Generali et al., 2015; Qin et al., 2018). Another study reported that low-dose paclitaxel significantly decreased cellular migration and invasion by downregulating S100A4, RhoA Cdc42 GTPase, MT1-MMP, and MMP9 (Cadamuro et al., 2016). Further, METRO administration of TOPO with pazopanib significantly altered tumor angiogenesis, cancer cell proliferation and apoptosis by regulating HIF1α and ABCG2 expression in metastatic triple-negative breast cancer (Di Desidero et al., 2015). In our previous studies, we reported the role of p21 in cell proliferation and survival (Aljuffali et al., 2011; Cardillo et al., 2013) after METRO-TOPO therapy.

Here, we determined gene expression patterns and the influence of METRO-TOPO treatment schedules in PCa. To characterize gene expression differences in the response to TOPO, we first performed transcriptome profiling of a panel of PCa cell lines representing androgen-sensitive/resistant, androgen receptor +/-, and those of different ethnicities. These treatment mediated gene expression signatures were then correlated with existing RNA-seq data from PCa patient cohort (Tuskegee University) and data from The Cancer Genome Atlas (TCGA). The goal was to determine if the observed gene signatures following METRO-TOPO were present in PCa patients/TCGA, and investigate their influence on PCa patient survival, as well as to identify any ethnic differences. The number and diversity of available PCa cell lines from AA patients is the major limitation of developing treatment specific (METRO) molecular signatures. Currently, there is only one PCa AA cell line (MDa-PCa-2b) available commercially. To overcome this limitation, we confirmed the expression of our treatment specific signatures (METRO) in three additional AA PCa cell lines (RC77, RC165, RC43) from our collaborator’s lab (Dr. Yates, Tuskegee University). Overall, our results provide insights into the gene pathway networks governing METRO-TOPO efficacy, as well as identifying pharmacogenomic signatures that may aid in personalizing treatment options for patients with PCa.

## Materials and Methods

### Chemicals and Reagents

Fetal bovine serum (FBS) and trypsin (0.25% w/v) were purchased from Hyclone (Thermo Fisher Scientific Inc. Rockford, IL). Topotecan (TOPO) was purchased from 21st Century Global E-Commerce Network (East Sussex, UK). Dimethyl sulfoxide (DMSO), sulforhodamine B (SRB), TRIS buffer, acetic acid, ECL western blotting substrate for chemiluminescence were obtained from Bio-Rad (Hercules CA, United States ). 3-(4,5-dimethylthiazol-2-yl)-2,5-diphenyltetrazolium bromide (MTT) and RNase A were purchased from Sigma-Aldrich Inc (St. Louis, MO). Mouse anti-human antibodies (MMP-9, MMP-1, Ang-2, VEGF, Maspin and c-Fos were purchased from Santa Cruz Biotechnology (uPA; H77A10, MMP-1; SB12e, Ang-2; F-1, VEGF; JH121, Maspin; E10 Santa Cruz, CA) and Cell Signaling Technology (PAI-1; D9C4, uPAR; D7X2N, MMP-9; D6O3H, c-FOs; 9F6, Danvers, MA). Goat anti-Rabbit IgG (H = L) Secondary antibody, HRP, was purchased from Cell Signaling Technology; 345,897 (Danvers, MA). *β*-actin was purchased from Sigma-Aldrich; A5316. All glass and plasticware were purchased from VWR (Radnor, PA).

### Cell Lines

Human PCa cell lines (PC-3, PC-3M, and DU145 representing metastatic, castration-resistant with nonendocrine differentiation (NEPC) (Yuan et al., 2007; Sun et al., 2009) and (LNCaP, C4-2B, VCaP, LaPC4, MDA-PCa-2b and 22Rv1 representing androgen-dependent or castration-sensitive (Hooker et al., 2019) were obtained from American Type Culture Collection (ATCC; Rockville, MD). DUTXR (DU145 taxanes resistance) was gifted by our collaborator Dr. Amit Kumar Mitra, Auburn University (Takeda et al., 2007). The cell lines were authenticated at source and tested for mycoplasma contamination. All cell lines are mycoplasma negative, and the other lines show consistent patterns. PC-3, PC-3M and MDA-PCa-2b cells were maintained in 10% (v/v) fetal bovine serum (FBS) supplemented in F-12K, DU145 in Eagle’s Minimum Essential Medium (EMEM), C4-2B, VCaP, and LaPC4 in Dulbecco’s Modified Eagle’s Medium (DMEM), LNCaP, 22RV1 and DUTXR in RPMI-1640 media with 10% FBS. All cells were maintained at 37°C, 21% O_2_ and 5% CO_2_ in a humidified cell culture chamber (Heracell™ VIOS 160i CO_2_; Thermo Scientific™). The RC77, RC165, RC43 cell lines were obtained from Dr. Yates; cell lines were developed from patients that self-identified as African American (Theodore et al., 2014). Cells were cultured in keratinocyte serum-free medium (KGM, Life Technologies, Carlsbad, CA).

### Patient Samples

RNA-seq data from prostate cancer patient samples was obtained from Dr. Clayton Yates. Details on patient samples have been analyzed and published previously (Theodore et al., 2014). Briefly, AA and CA patient tissue biopsies were obtained along with patient-matched normal adjacent tissues and used for gene expression analysis. No significant differences were detected between the two groups with respect to tumor content. As mentioned in our earlier publication, Institutional Review Board approvals were obtained from the Institutional Review Boards of Tuskegee University (IRB 00001137), St. Frances Hospital and Medical Center, Hartford CT, and UAB (Theodore et al., 2014).

### Metronomic and Conventional Treatment Protocols

PCa cell lines were seeded at 4 × 10^3^ cells in 96 well plates with appropriate media with 10% (v/v) FBS supplemented (Fontana et al., 2009). Plates were incubated for 24 h before media change and replacement with serum-supplemented media containing TOPO (0.04–10,000 nM). For CONV treatments, plates were then incubated at the same conditions for an additional 48 or 72 h. Whereas, for METRO treatment media was removed at 24 and 48 h after initial dosing, and cells were exposed to freshly prepared serum-supplemented media containing TOPO (0.04–10,000 nM). The media for control cells was changed daily and did not contain drug (Fontana et al., 2009). All experiments were performed in triplicate with five replicate wells for each concentration. Cell growth and cytotoxicity was assessed at each time point.

### Assessment of *in vitro* Cell Growth and Cytotoxicity

The effect of TOPO treatment on prostate cell growth and cytotoxicity was conducted by measuring total protein (SRB), mitochondrial enzyme activity (MTT), and examination of cellular morphology. SRB and MTT staining were performed at 48 and 72 h post initial TOPO exposure as described previously (Fontana et al., 2009). Absorbance was measured at 490 nm using a Synergy 3 Multi-Mode Microplate Reader (BioTek, Winooski, VT). The effect of drug exposure was determined by constructing cytotoxicity (growth) curves (*n* = 5 wells/group), percent change relative to untreated controls was calculated at each drug concentration. Half-maximal inhibitory drug concentration (IC_50_) values were estimated by nonlinear regression using a sigmoidal dose-response equation (variable slope - three parameters).

### Apoptosis Assay (Caspase 3/7)

Caspase-3/7 activation was measured using the Caspase-Glo 3/7 assay (Caspase-Glo^®^ 3/7 Assay - Promega Corporation) according to the manufacturer’s protocol. Briefly, a total of 2×10^4^ cells/well were seeded into 96-well plates (triplicates) and treated at the estimated 72 h MTT IC_50_ (METRO and CONV). Caspase-Glo 3/7 reagent was added and incubated for 2 h, and luminescence was measured using a Synergy 3 plate reader. We normalized the level of apoptosis in each treatment group with the control group (no drug treatment or baseline caspase 3/7 assay luminescence) for each cell line.

### Assessment of Cellular and Nuclear Morphology

Cell morphology was assessed using phase-contrast microscopy. PC-3 and LNCaP cells were seeded (4 × 10^3^) in 6-well plates and exposed to TOPO at the estimated IC_50_ for 72 h (MTT) for each treatment protocol (CONV and METRO). Three areas with approximately equal cell densities were identified in each well, and images were captured with a Nikon AZ100 stereo-fluorescent microscope mounted with a Nikon Digital Sight DS-QiMc camera utilizing NIS-Elements image analysis software (Nikon, Melville, NY). Images were recorded in the modes of bright field and phase contrast at ×20 and ×40 magnifications. Further, images were analyzed using ImageJ (NIH, v. 1.46j, imagej.nih.gov).

### Scratch Assay

Scratch assays were performed by creating a “scratch” in a cell monolayer followed by capturing the images at the beginning and regular interval (0, 48 and 72 h for METRO and CONV treatments) during cell migration to close the scratch and comparing the images to quantify the migration rate of the cells. Briefly, PC-3 cells were plated in 6-well plates at 1 × 10^5^ cells/well and incubated for 48 h to 90% confluency. The monolayer was scratched with a p200 pipette tip at the center of the well. F-12K culture medium, supplemented with 10% FBS, containing the vehicle (0.5% DMSO) was added to the cells in the control wells, and micrographs obtained at 0, 48, and 72 h. The effect of CONV and METRO dosing of TOPO, at their respective IC_50_, were applied to the cells in the respective wells, and micrographs of the wound areas were obtained at 0, 48, and 72 h using an EVOS FL digital cell imaging system (Thermo Fisher Scientific, Inc.). Images were recorded in the modes of bright field and phase contrast at ×20 and ×40 magnifications. The initial wound area (at 0 h) and the “gap area” were measured at 48 and 72 h with ImageJ software.

### RNA Isolation

Total RNA was isolated from cultured cells and patient samples using standard RNA extraction kits (RNeasy Kits–QIAGEN). RNA concentration and integrity were estimated by a NanoDrop 2000 UV-Vis spectrophotometer (Thermo Scientific, United States ), Qubit^®^ 2.0 Fluorometer (Invitrogen, Carlsbad, CA, United States ), and Agilent 2,100 Bioanalyzer (Applied Biosystems, Carlsbad, CA, United States ).

### Targeted Gene Expression Analysis

Gene expression in PC-3 and LNCaP cells were assessed at the calculated IC_50_ of 48 and 72 h TOPO treatment (CONV vs METRO) using the RT^2^ Profiler™ PCR Array Human Cancer Pathway Finder (PAHS-033A). The qRT-PCR analysis was performed using a LightCycler^®^ 480 II Real-Time PCR System (Roche Applied Science, Indianapolis, IN). Three housekeeping genes (GAPDH, ACTB, HGDC), reverse transcription control (RTC - Control RNA + primer) and positive PCR control (PPC) were used for assessing PCR efficacy. Gene expression was calculated by ΔΔCt method. Gene expression in treatment groups were normalized to corresponding controls (no drug treatment) and differentially expressed genes (DEGs) were identified.

### Next-Generation RNA Sequencing (RNAseq)

Details on NGS-based RNA sequencing and data availability have been published earlier (Theodore et al., 2014). Briefly, Total RNA from patients and cell lines were taken and ribosomal RNA was removed with Ribo-Zero™ Gold kits (Epicenter, Madison, WI, United States ) by the manufacturer’s recommended protocol. Then, the RNA was fragmented and primed for the first-strand synthesis using the NEBnext First-Strand synthesis module (New England BioLabs Inc. Ipswich, MA, United States). The second-strand synthesis was performed with the NEBnext Second Strand synthesis module. Following this, the samples were added to a standard library preparation protocol using a NEBNext^®^ DNA Library Prep MasterMix Set for Illumina (Theodore et al., 2014). Library quantity and quality were assessed, and paired-end RNA sequencing was performed with an Illumina HiSeq2500 sequencer (Illumina, Inc. San Diego, CA, United States).

### Ingenuity Pathway Analysis (IPA)

IPA (QIAGEN) was used to identify the most significantly affected 1) canonical pathways predicted to be activated or inhibited; 2) upstream regulator molecules like transcription factors, may be causing the observed gene expression changes; 3) downstream effects and biological processes that are increased or decreased; 4) predicted causal networks from our differential mRNA expression analysis (Krämer et al., 2014).

### Patient Gene Expression Data

Processed, high-quality RNAseq data PCa patients were obtained from Dr. Clayton Yates’ lab (Tuskegee University) (Theodore et al., 2014). Details on RNA sequencing data availability have also been published earlier (Theodore et al., 2014). These reads were aligned to the reference genome (GRCh37 assembly) using HISAT2 (version 2.0.4), assembled into potential transcripts, and gene expression levels were quantified using Stringtie (version 1.3.3).

### In silico Evaluation Using The Cancer Genome Atlas (TCGA) Database

The mRNA expression of PCa patients was extracted from The Cancer Genome Atlas (TCGA) Data Portal (cancergenome.nih.gov). 717 PCa patients (500 prostate adenocarcinoma) and (217 cases from the Foundation Medicine Adult Cancer Clinical Dataset) had mRNA expression data available. R-based pipeline was created, and UALCAN, an interactive web-portal was used to perform data download, processing, and in-depth analysis of gene expression data files from TCGA’s Genomic Data Commons (GDCs) server and retrieve transcriptome data on target candidate pathway genes from the prostate expression data matrix containing >60,000 transcripts (Chandrashekar et al., 2017).

### Western Blotting

PCa cells were seeded and exposed to TOPO at the estimated 72 h MTT IC_50_ for each treatment protocol (CONV and METRO). Post-treatment cells were lysed in cell lysis buffer (Thermo Scientific™ RIPA Buffer). Equal amount of protein loaded onto 4–15% Criterion™ TGX Stain-Free™ Precast Gels. Proteins will be separated under reducing conditions and then transferred to a PVDF membrane using a Trans-Blot Turbo Mini transfer pack from Bio-Rad (Hercules CA, United States ). Nonspecific binding was limited by incubating the membrane in blocking buffer (2.5% (w/v) casein, pH 7.6, 150 mM NaCl, 10 mM TRIS-HCl, and 0.02% sodium azide). Membranes were incubated with primary antibodies for target protein (1:1000) for overnight and then with the appropriate secondary antibody (1:10,000) at room temperature. Immunoreactivity was detected by Pierce ECL Western Blotting substrate (Bio-Rad, CA). Images were captured and quantify by Gel Doc™ EZ Gel Documentation System and ImageLab™ Software (Hercules CA, United States ). Densitometry analysis was performed using standard image analysis software ImageJ.

### Bioinformatics and Statistical Analysis

All statistical analyses were performed using R statistical programming package (version 3.6.2) and GraphPad Prism (version 7.0). Gene expression profiling (GEP) data were analyzed using Partek Genomics Suite v7.0 software to identify DEGs. Owing to the small sample size, we use limma, an empirical Bayesian method, to detect the DEGs and obtain *p* values and further provided False discovery rate (FDR) based on the *p*-value using Benjamini–Hochberg procedure. All tests were a two-sided and *p* < 0.05 was considered statistically significant. For analysis of RNAseq data obtained from EA/AA patient cohort, FDR adjusted *p*-values were used to determine significant associations.

## Results

### *In vitro* Growth

**FIGURE 1:**
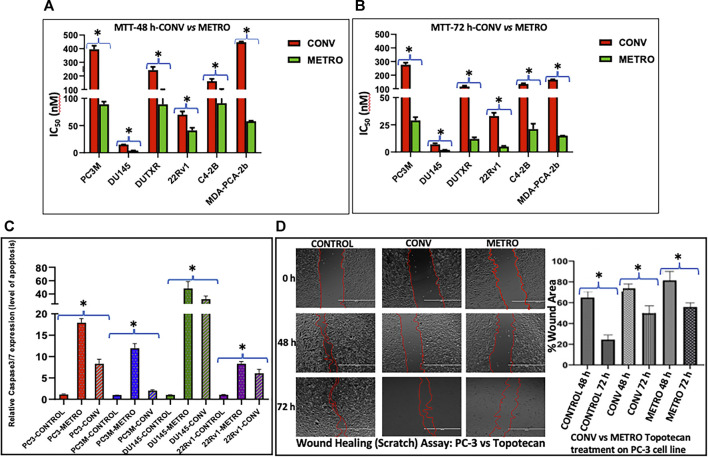
Effect of topotecan on the growth of prostate cells *in vitro* following Conventional and Metronomic dosing. **(A, B) *In vitro*** cytotoxicity. cytotoxicity was assessed following 48 and 72 h of CONV or METRO treatment in PC-3M, DU145, DUTXR, 22Rv1, C4-2B and MDA-PCA-2b cell lines using mitochondrial activity (3-(4, 5-Dimethylthiazol-2-yl)-2,5-diphenyltetrazolium bromide or MTT assay) at increasing drug concentrations (Significant *p* value * = *p* ≤ 0.05). **(C)** Apoptosis assay. Level of caspase3/7 enzyme activity measured after METRO and CONV treatment of TOPO for 72 h; METRO-TOPO treatment exhibited higher apoptosis than CONV-TOPO treatment mCRPC/NEPC cell line (PC-3, PC-3M, DU145) and androgen-sensitive 22Rv1. Representative data is shown and similar data was collected for all the cell lines (Significant *p* value * = *p* ≤ 0.05). **(D)** Cell migration. Wound healing (Scratch) assay performed by measuring cell migration, invasion and growth after METRO and CONV treatment of TOPO for 48 and 72 h. METRO-TOPO treatment exhibited reduce wound healing than CONV-TOPO treatment mCRPC cell line (PC-3) (Significant *p* value * = *p* ≤ 0.05).

The effect of TOPO administration as CONV and METRO on prostate cancer cells were assessed by measurement of MTT (Figures 1A,B) and SRB (Supplementary Figure S1A) absorbance after 48 h and 72 h treatments. Briefly, for CONV treatment, cells were treated with increasing concentrations of (0.04–10,000 nM) at 24 h following incubation, while for METRO, cells were exposed to freshly prepared serum-supplemented media containing TOPO (0.04–10,000 nM) at 24 and 48 h after initial dosing, and cells were exposed to freshly prepared serum-supplemented media containing TOPO (0.04–10,000 nM (Aljuffali et al., 2011). METRO exposure to TOPO in metastatic, androgen-insensitive, NEPC (EA: PC-3M, DU145 and DUTXR) and metastatic, androgen-sensitive (EA: C4-2B, 22RV1and AA: MDA-Pca-2b) cells resulted in significant (*p* ≤ 0.05) time-dependent decreases in the IC_50_ value, which reflects increased sensitivity after 48 and 72 h compared to CONV treatment (Table 1). Comparison to CONV, METRO dosing for 72 h decreased the IC_50_ for MTT by 9.6, 4.0, 9.7, 6.2, 6.4, and 10.7- fold in PC-3M, DU145, DUTXR, 22RV1, C4-2B and MDA-PCa-2b, respectively, as shown in Table 1. Further, TOPO METRO was more potent in AA cell lines (MDA-Pca-2b) by decreasing the IC_50_ by 11-fold (165.93 vs 15.47 nM by MTT) after 72 h. Similar results were observed after 48 and 72 h of treatment and by the SRB assay as shown by a Supplementary Figure S1A).

**TABLE 1:** *In vitro* chemo-sensitivity (IC_50_ values for MTT and SRB assays) data presented (nM) as mean ± SEM of at least three independent studies (*n* = 5/study).

Cell line	PC-3M	DU145	DUTXR	22Rv1	C4-2B	MDA-PCA-2b
Ethnicity	Caucasian	Caucasian	Caucasian	Caucasian	Caucasian	African american
Characteristic	Androgen-independent	Androgen-independent	Androgen-independent	Androgen-sensitive	Androgen-independent	Androgen-sensitive
IC_50_-MTT-CONV-48 h	395 ± 27	15 ± 0.23	242 ± 24	70 ± 6	161 ± 17	447 ± 5
IC_50_-MTT-METRO-48 h	89 ± 5	4.0 ± 0.23	89 ± 18.1	41 ± 5	91 ± 16	58 ± 0.8
Fold-Change	4.42*	3.68*	2.72	1.77	1.76	7.67
IC_50_-SRB-CONV-48h	1340 ± 160	38 ± 4	142 ± 13	285 ± 15	196 ± 12	
IC_50_-SRB-METRO-48 h	499 ± 1	4.0 ± 0.74*	44 ± 10.7	134 ± 14	83 ± 7	
Fold-Change	2.68	9.64*	3.23*	2.13	2.36	
IC_50_-MTT-CONV-72 h	276 ± 15	7 ± 1	116 ± 6.15	33 ± 3	135 ± 5	166 ± 2.46
IC_50_-MTT-METRO-72 h	29 ± 3*	2.0 ± 0.07*	12 ± 1.43	5.0 ± 0.66*	21 ± 5	15 ± 0.11
Fold-Change	9.60*	3.96*	9.66*	6.23*	6.42*	10.73
IC_50_-SRB-CONV-72 h	371 ± 57	9.0 ± 0.18	78 ± 5.10	136 ± 10	289 ± 3	
IC_50_-SRB-METRO-72 h	28 ± 3	4.0 ± 0.06*	21 ± 6.96	18 ± 2*	36 ± 3.4	
Fold-Change	13.2*	2.57*	3.71*	7.64*	8.02	

METRO values with (*) are significantly (*p* ≤ 0.05) different in comparison to conventional (CONV) treatment.

### Apoptosis

Schedule-dependent (METRO *vs* CONV) effects on apoptosis were determined by measuring caspase-3/7 activity following treatment. METRO dosing of TOPO induced apoptosis in every cell line (PC-3–2.5, PC-3M-5.9, DU145–1.4 and 22RV1 1.6-fold) tested as compared to cells with CONV treatment (Figure 1C). Consistent with results from growth assays, apoptosis assay showed METRO TOPO to be a more effective schedule as compared with CONV TOPO.

### Cell Morphology

Cell morphology studies, as shown in Supplementary Figure 1B, confirmed the differences in the IC_50_ values (MTT, and SRB staining) as well as apoptosis assays. PC-3 and LNCaP cells exposed to METRO dosing regimens showed greater decreases in cellular density (3.6 and 6.2-fold) compared with CONV dosing.

### Cell Migration

PC-3 cells were exposed to TOPO CONV and METRO IC_50_ at 48 and 72 h to examine the effect of different dose regimen on cell migration as measured using the scratch/wound healing assay. Results revealed that METRO-TOPO treatment had a greater effect (∼2-fold) in reducing cell migration in prostate cancer cell lines (*p* < 0.05) (Figure 1D).

### Targeted Gene Expression Analysis

**FIGURE 2:**
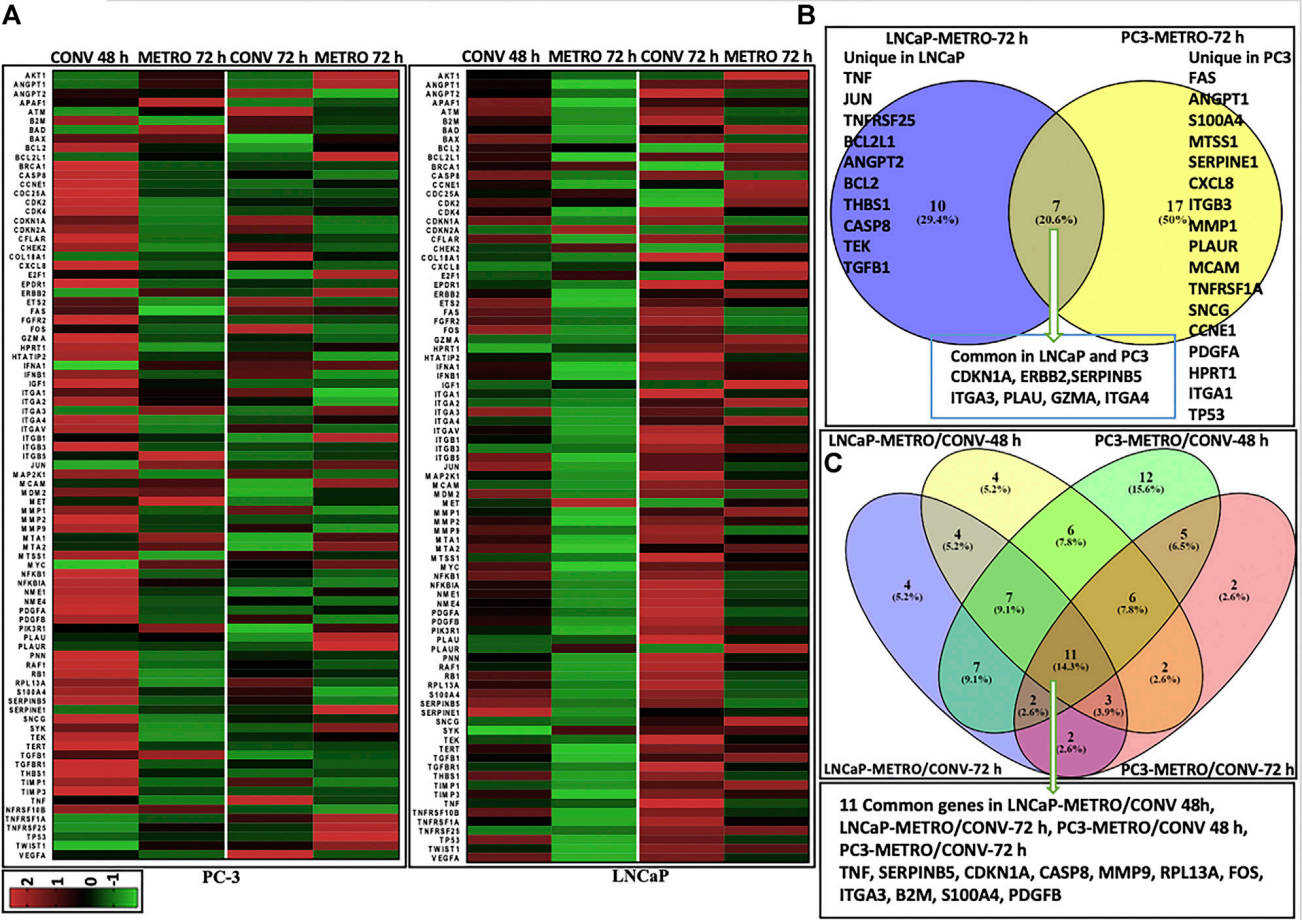
Differential gene expression results Heatmaps representing differential gene (mRNA) expression between CONV *vs* METRO Topotecan treatment in PC-3 and LNCaP cell lines. Each row corresponds to one differentially expressed gene (DEG) ordered alphabetically. Each column represents a single representative sample. Log_2_ ratios are depicted in a color scale where red represents upregulation and green represents downregulation (fold change cut-off value > 2). **(A)** DEGs for CONV and METRO TOPO treatments for I) PC-3 and II) LNCaP cell lines. Gene expression levels were assessed by RT-PCR (RT Profiler™ PCR Array Human Cancer PathwayFinder (PAHS-033A) array, QIAGEN; total number of genes (*n*) = 91; including three control; housekeeping genes–GAPDH, ACTB, HGDC). Gene expression was calculated by ΔΔCt method: i) Delta Ct = Ct Target gene- Ct Control gene (Beta actin; ACTB). ii) Expression in treatment groups were normalized to corresponding control (no drug treatment for both PC-3 and LNCaP cell line. Venn diagrams represent unique and common DEGs for metronomic treatment in PC-3 and LNCaP. **(B)** Post METRO treatment unique and commonly expressed gene profile. I) 17 Unique DEGs for METRO treatment in PC-3; 10 Unique DEGs for METRO treatment in LNCaP; 7 genes (CDKN1A, ERBB2, SERPINB5, ITGA3, PLAU, GZMA and ITGA4) were expressed commonly in both cell lines (PC-3 and LNCaP) following METRO TOPO treatment. II) Top DEGs were (*n* = 11) TNF, SERPINB5, CDKN1A, CASP8, MMP9, RPL13A, FOS, ITGA3, B2M, S100A4 and PDGFB when all the treatment groups were considered together for all the cell lines (CONV and METRO at 48 and 72 h treatment regimen in PC-3 and LNCaP).

Next, we performed targeted gene expression profiling (GEP) to identify and compare changes in gene expression following TOPO METRO *vs* CONV treatments in androgen-independent *vs* androgen-sensitive cell lines. GEP data from each treatment group and treatment time point was normalized to baseline gene expression for the corresponding cell line and heatmaps were generated (Figure 2A). Top differentially expressed genes/DEGs were identified for METRO *vs* CONV TOPO treatments in PC-3 and LNCaP at 48 (Supplementary Table S1) and 72 h (Table 2).

**TABLE 2:** Top differentially expressed genes (DEGs) (fold change compared to untreated) following 72 h of CONV- or METRO-TOPO treatment in PC-3 (mCRPC/NEPC) and LNCaP (metastatic, castration-sensitive PCa) cell lines. Fold change cut-off value is > 2. CDKN1A, FOS, TNF and SERPINB5 were expressed in both treatment groups but there was variation in expression levels as these genes were downregulated in the METRO treatment group and upregulated in the CONV group.

Gene	PC-3		LNCaP	
	72 h topo-conv vs control	72 h metro-topo vs CONV-TOPO	72 h CONV-TOPO vs control	72 h metro-topo vs CONV-TOPO
CDKN1A	13.1	−7.5	14.7	−6.6
FOS	9.5	−9.1	2.2	−2.1
IFNB1	4.3	−4.7	NA	NA
TNF	3.7	−2.9	20.4	−11
VEGFA	3.2	−2.5	6.8	NA
MMP1	2.8	−5.6	NA	NA
CDKN2A	2.2	−2.8	NA	NA
SERPINB5	2.1	−3.7	13	−7.5
ANGPT2	2	NA	7.2	−4.3
PLAU	NA	NA	4	−2.5
FGFR2	NA	NA	3.7	−3
TEK	NA	NA	3.5	−2.3
MDM2	NA	NA	3.4	−2.5
PDGFA	NA	NA	3.3	−2.4
RPL13A	NA	NA	2.8	−2.3
MMP9	NA	−2	2	−2.5
CDC25A	NA	NA	−2.5	2.4
BRCA1	NA	NA	−3.6	2.5
PLAUR	NA	NA	−4	3.2
E2F1	NA	2.2	−4	4.3

As shown in Table 2, the top 4 DEGs between pre- (untreated; 0 h) vs post-treatment (72 h), irrespective of androgen-sensitivity or treatment type (CONV *vs* METRO) were CDKN1A, FOS, TNF and SERPINB5 (Supplementary Figure S2). On the other hand, for METRO treatment only, a total of 34 genes were differentially expressed at 72 h (*p* < 0.05, fold-change>2). Among these, 17 were unique to PC-3, while 10 were unique to LNCaP. Seven 7) of these DEGs were common to both cell lines signifying a METRO-TOPO-specific GEP signature for PCa (Figure 2B I). Ovrall, the GEPs between treatments (METRO and CONV) identified eleven (*n* = 11) candidate biomarkers (Figure 2B II). The top five genes were SERPINB5, CDKN1A, TNF, FOS, and ANGPT1 (Table-2).

### Ingenuity Pathway Analysis (IPA)

**FIGURE 3:**
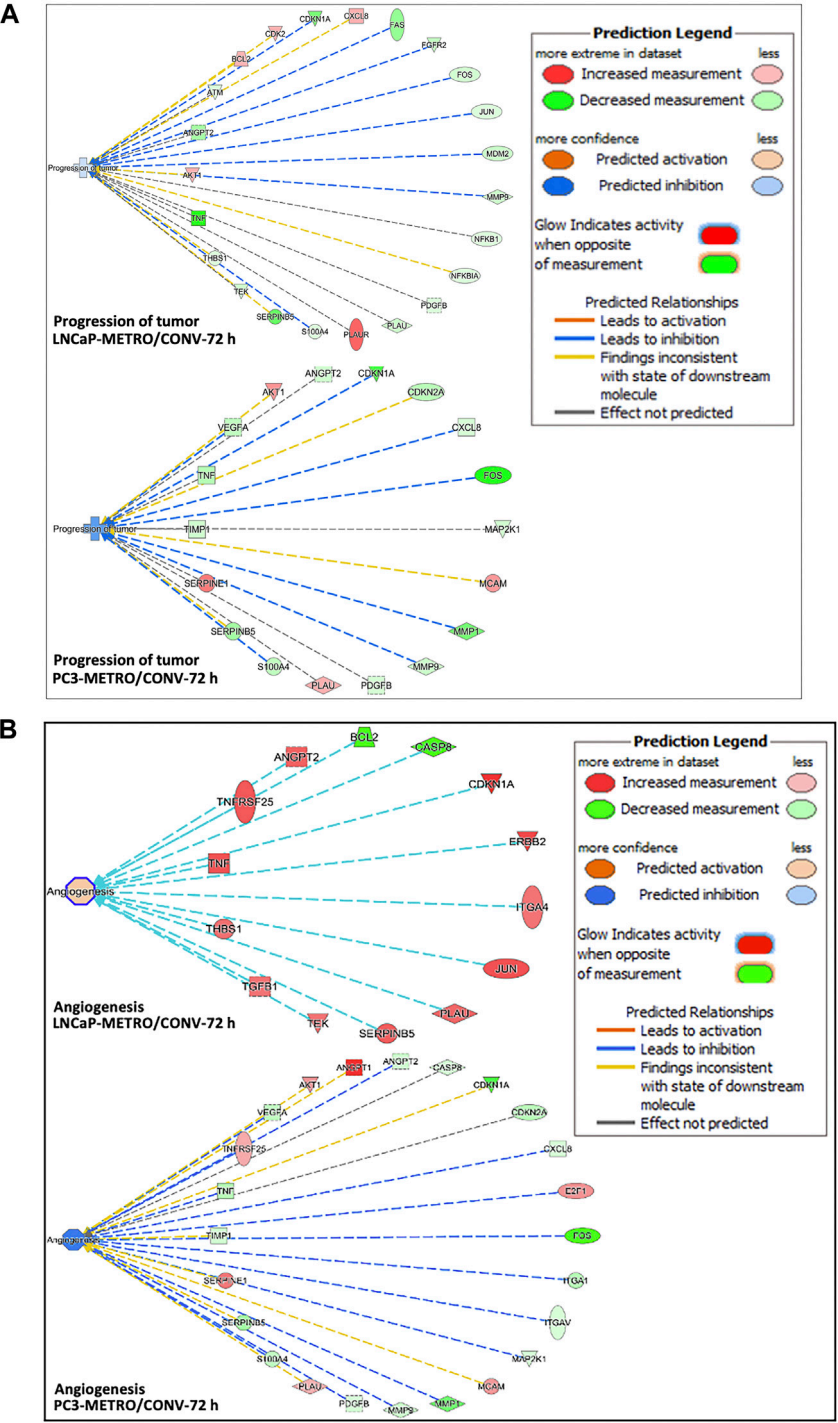
Ingenuity pathway analysis predictions. **(A)** IPA predicted higher tumor progression inhibition in PC-3 compared to the LNCaP cell line following TOPO METRO at 72 h treatment. FOS, CDKN1A and SERPINB5, PLAUR, TNF and BCL2 are key genes in the tumor progression pathway that were expressed differentially between the two different treatment regimens, which predicted higher tumor progression inhibition for METRO-TOPO treatment. **(B)** Anti-angiogenesis (inhibition of angiogenesis) pathway. where METRO treatment resulted in higher downregulation in PC-3 compared to the LNCaP cell line for TOPO METRO at 72 h treatment.

IPA analysis performed based on the DEGs associated with METRO-TOPO compared to CONV treatment predicted inhibition of tumor progression and angiogenesis as the key pathways for METRO-TOPO. This involved downregulation of SERPINB5, S100A4, MMP9, FOS, CDKN1A, TNF, and upregulation of, PLAU, CASP8, TNFRSF25 and BCL2 (LNCaP) (Figure 3A). Additionally, IPA predicted greater anti-angiogenic activity in PC-3 compared to LNCaP cell line for TOPO METRO treatment (Figure 3B). MMP1 was predicted by IPA as one of the top METRO-TOPO treatment ‘upstream regulators’ for LNCaP and PC-3 cell lines based on differential regulation of many important genes (BAX, TNF, SERPINEB5, S10000A4, PLAU, MMP9, FOS, CXCL8, CDKN1A) which in turn were predicted to result in dysregulation of cell proliferation, migration, metastasis, invasion and angiogenesis. Together, these may explain the potency of METRO-TOPO treatment in PCa (Supplementary Figure 3D). Interestingly, although TOPO is not an FDA approved drug for PCa, IPA analysis predicted PCa signaling (*p* = 1.40E-14) and PTEN (*p* = 1.53E-13) signaling pathways as targets for METRO TOPO treatment (Supplementary Table S2). Further, IPA also predicted small cell lung cancer as a top disease target for TOPO treatment, for which it is already an FDA-approved drug (*p* = 7.55E-17). IL-8 and p53 signaling pathways were also predicted as targets for METRO-TOPO uniquely in LNCaP cells (Supplementary Figure S3A–C).

### Correlation of Top DEGs in a PCa Patient Cohort

**FIGURE 4:**
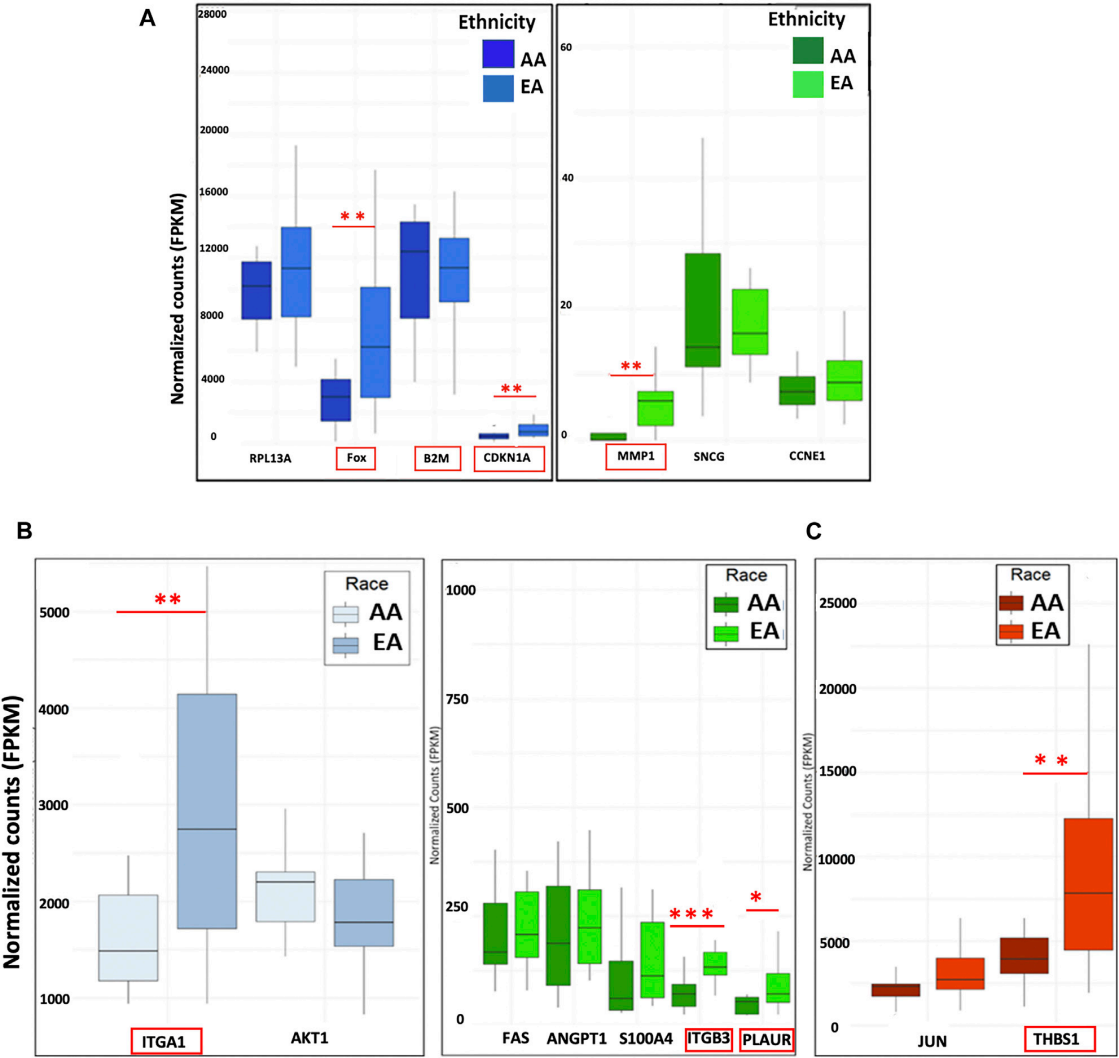
Correlation of METRO-TOPO gene expression signatures in the PCa patient cohort. **(A)** Top DEGs for METRO-TOPO dosing were also differentially expressed in prostate cancer patient cohort (*p* * = *p* ≤ 0.05, ** = *p* ≤ 0.01, *** = *p* ≤ 0.001). FOS, CDKNA1 and MMP1 expression is lower in AA patients compared to EA patients, whereas B2M expression showed trends of higher expression in AA patients compared to EA patients. **(B)** METRO-TOPO genes unique to AI-mCRPC (*p* * = *p* ≤ 0.05, ** = *p* ≤ 0.01, *** = *p* ≤ 0.001). Expression of ITGA1, ITGB3 and PLAUR was lower in AA compared to EA patients, whereas PLAUR expression was higher in AA patients. C) METRO-TOPO gene signature unique to castration-sensitive PCa (** = *p* ≤ 0.01). THBS1 expression was lower in AA compared to EA patients (FPKM is the Fragments Per Kilobase of transcript per Million mapped reads. In RNA-Seq, the relative expression of a transcript is proportional to the number of cDNA fragments that originate from the gene.

Next, we assessed the top DEGs for METRO-TOPO using GEP data on PCa patient tumors. FOS, B2M, CDKNA1, and MMP1 were differentially expressed (Figure 4A). The expression of CDKNA1 and MMP1 were lower, while B2M was expressed at a higher level in AA compared to EA patients. Further, ITGA1, and ITGB3, which were unique for METRO-TOPO in metastatic, androgen-insensitive, NEPC had a significantly lower expression in AA, while PLAUR expression was higher in AA compared to EA PCa patients (*p* ≤ 0.05) (Figure 4B). THBS1—another DEG unique to metastatic, androgen-insensitive, NEPCs for METRO-TOPO, was also significantly downregulated in AA *vs* EA PCa patient cohorts (*p* ≤ 0.01) (Figure 4C).

### *In silico* Correlation of the Top DEGs Using TCGA PRAD Patient Cohort

**FIGURE 5:**
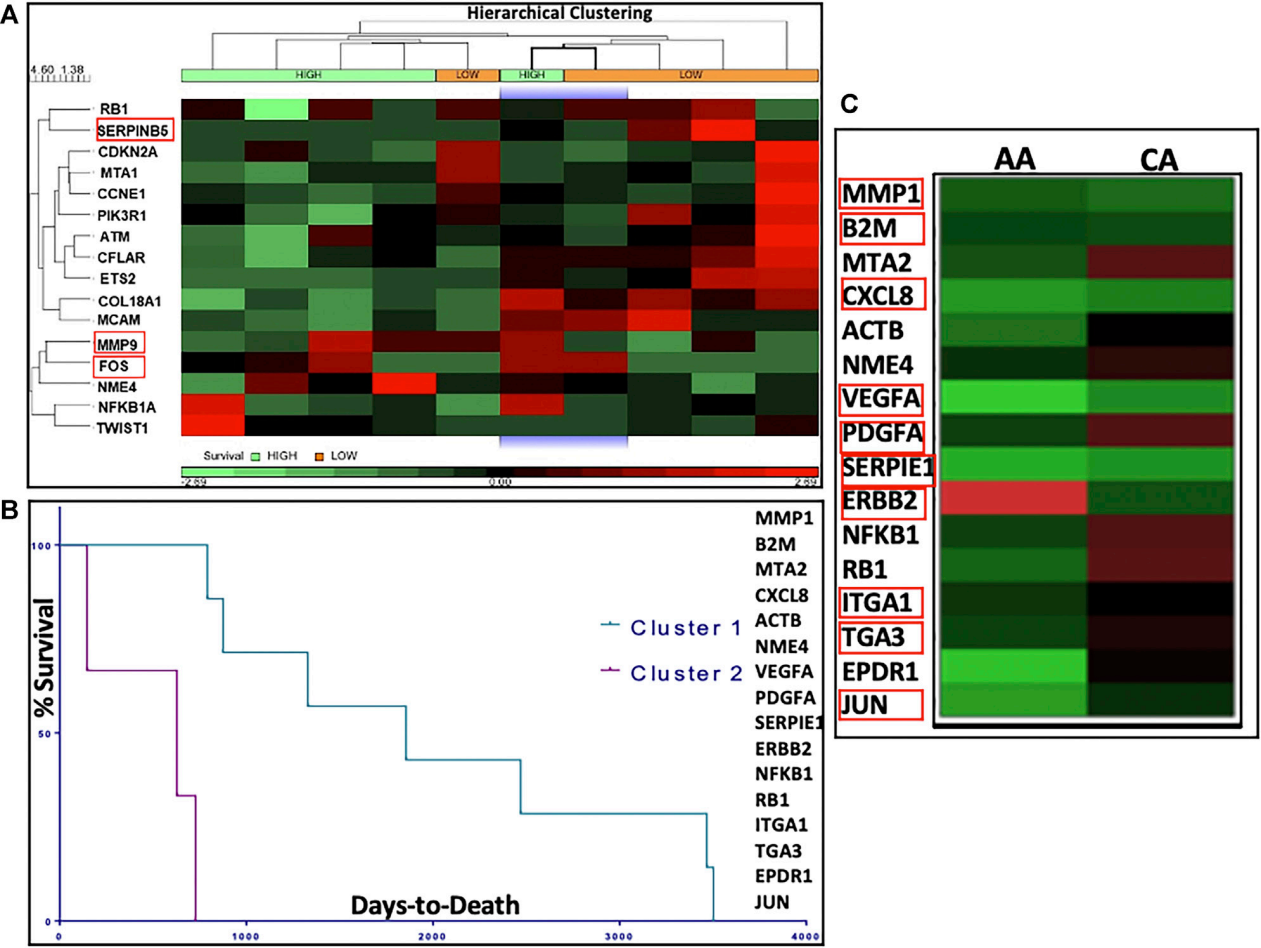
In silico correlation of METRO-TOPO treatment signatures using The Cancer Genome Atlas (TCGA) database. **(A)** Heatmap represents differential gene expression of METRO-TOPO genes between High vs Low survival prostate cancer (PRAD) subgroups in TCGA database. Hierarchical clustering (HC) analysis was used to group row-level (genes) and column-level (patient samples) based on differential gene expression. Differential expression of MMP9, FOS and SERPINB5 were significantly associated with patient survival. **(B)** Kaplan-Meier plots showed stratification in survival among the TCGA prostate cancer patient clusters. K-means clusters (class = 2) were generated based on the highly differentially expressed genes (DEGs) for METRO-TOPO treatment. **(C)** Heatmap represents differential gene expression between AA (African American) and CA (Caucasian American) men for the top DEGs (MMP1, B2M, CXLC8, VEGF, PDGFA, SERPINE1, ERBB2, ITGA1, ITGA3 and JUN).

We performed *in silico* analysis of the top DEGs using patient GEP data available in the TCGA database. We explored the following groups: 1) All men with PCa; 2) AA *vs* EA men with PCa and 3) high *vs* low survivors. Differential gene expression between high *vs* low survival PCa subgroups is depicted by the heatmap in Figure 5A. Results showed that the top DEGs for METRO-TOPO in PCa cell lines models, including SERPINB5, CDKN2A, and MMP9, were also correlated significantly (*p* < 0.05) with TCGA PCa patient’s survival. Additionally, we used TCGA databases to derive K-means clustering to show the top genes expression profile that are beneficially associated with prostate cancer patient survival. Kaplan-Meier plots were derived following K-means clustering (class = 2), showing stratification in survival among TCGA PCa patient clusters based on the top DEGs for METRO-TOPO treatment (Figure 5B II). Among these, the downregulation of MMP9, FOS, and SERPINB5 was correlated significantly (*p* < 0.05) with high patient survival (Supplementary Table S4). Further, expression of these top genes was also associated with patient survival specific to AA (Supplementary Table S3). Finally, TOPO METRO specific genes - MMP1, B2M, CXLC8, VEGF, PDGFA, SERPINE1, ERBB2, ITGA1, ITGA3, and JUN were expressed differentially between EA *vs* AA PCa patients (Figure 5C).

### Correlation of GEP Data in an Expanded Set of AA vs EA PCa Cell Lines

**FIGURE 6:**
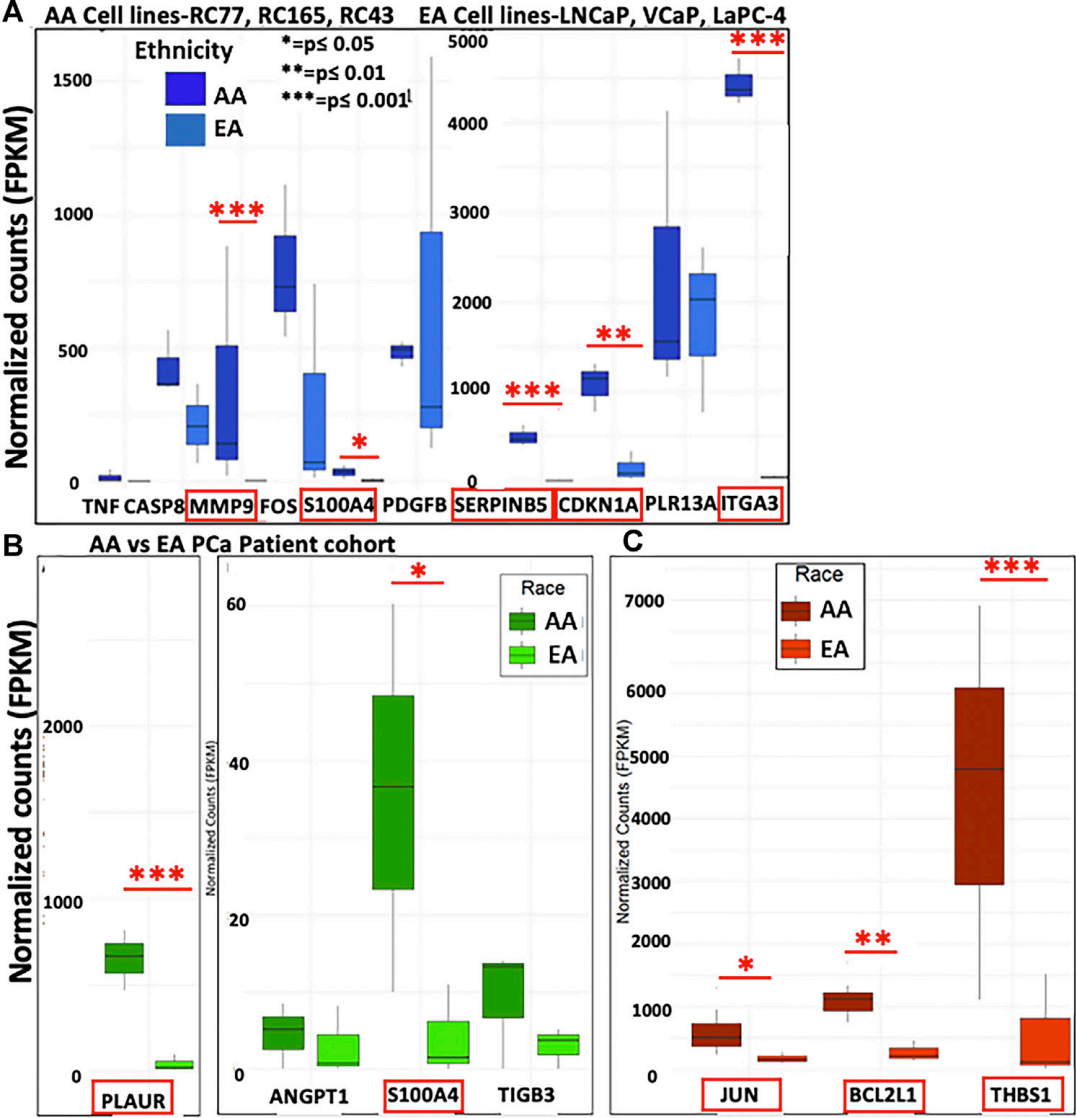
Determination of METRO-TOPO gene expression signatures in cell lines representing EA *vs* AA patients. **(A)** Top DEGs for METRO-TOPO dosing (SERPINB5, CDKN1A, MMP9, ITGA3 and S100A4) were differentially expressed significantly in AA cell line vs EA cell lines (RC77, RC165, RC43 vs LNCaP, VCaP, LaPC4) (*p* * = *p* ≤ 0.05, ** = *p* ≤ 0.01, *** = *p* ≤ 0.001). Expression of all these genes were higher in AA compared to EA cell lines. **(B)** METRO-TOPO genes unique to AI-mCRPC (*p* * = *p* ≤ 0.05, ** = *p* ≤ 0.01, *** = *p* ≤ 0.001). Expression of PLAUR and S100A4 was higher in AA compared to EA cell lines. **(C)** METRO-TOPO gene signature unique to castration-sensitive PCa (*p* * = *p* ≤ 0.05, ** = *p* ≤ 0.01, *** = *p* ≤ 0.001). JUN, BCL2L1 and THBS1 expression levels were higher in AA cells compared to EA-derived cell lines.

Our TCGA analysis demonstrated that several *in vitro* METRO-TOPO gene signatures including SERPINB5, MMP9, and FOS were correlated with patient survival and varied significantly (*p* < 0.05) between AA *vs* EA PCa patients. However, this analysis was based on one AA PCa cell line and limited number of AA patients within the TGCA. To overcome this limitation, we further compared our signature with gene expression profiles from patient derived PCa cell lines from AA (RC77, RC165, RC43) and EA (LNCaP, VCaP, LaPC4). In untreated cells, we observed increased expression of SERPINB5, CDKN1A, MMP9, ITGA3, and S100A4 in AA cell lines compared to EA cell lines (Figure 6A). Further, the METRO-TOPO DEGs unique to PC-3 were PLAUR and S100A4, and LNCaP were JUN, BCL2L1, and THBS1, were also found to be expressed at enhanced levels in AA compared to EA-derived cell lines (*p* ≤ 0.05) (Figures 6B,C).

### Immunoblotting to Confirm Protein Level Changes

**FIGURE 7:**
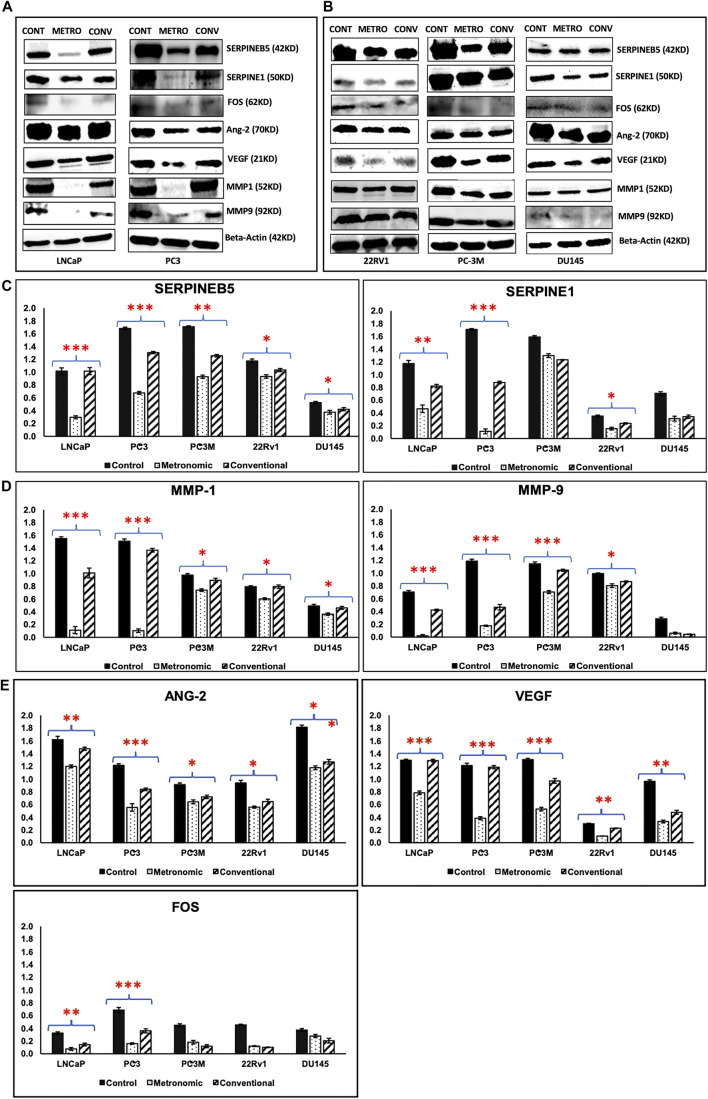
Western blotting/Immunoblot analysis. Western blotting analysis results of proteins representing top DEGs for METRO-TOPO treatment in castration-resistant and androgen-sensitive cell lines. Beta Actin was used as a control (housekeeping) gene. **(A)** Metastatic, androgen-sensitive LNCaP, mCRPC/NEPC PC-3; **(B)** mCRPC/NEPC PC-3M, DU145 and metastatic, androgen-sensitive 22RV1. **(C, D, E)** Densitometry plots METRO-TOPO dosing-associated DEGs compared to CONV and Control. SERPINB5, SERPINE1, FOS, ANG-2, VEGF, MMP1 and MMP9 were significantly downregulated in METRO-TOPO vs CONV-TOPO in PCa cell lines (*p ≤ 0.05, **p ≤ 0.01, ***p ≤ 0.001).

Based on our expression data and *in silico* analyses, we confirmed protein expression of the top METRO-METRO DEGs *via* immunoblotting in PCa cell lines. We observed 1) SERPINEB5 and SERPINE1 were downregulated (RNA-seq) in METRO-TOPO; 2) FOS. iii) MMP1 and MMP9—identified as the most important genes by IPA; and 4) ANGPT2 and VEGF. Western blotting results show that protein expression of these top DEGs were concurrently higher in castration-resistant PC-3, and PC-3M compared to hormone-sensitive LNCaP, and 22RV1 (Figure 7). Furthermore, consistently greater downregulation of these top DEGs was observed following METRO-TOPO treatment compared to CONV-TOPO treatments. Figures 7A–E shows that densitometry plots for the proteins encoded by SERPINB5, SERPINE1, FOS, ANG-2, VEGF, MMP1, and MMP9 showed significant differential expression between METRO-TOPO *vs* CONV-TOPO dosing in PCa cell lines (*p*-values * = *p* ≤ 0.05, ** = *p* ≤ 0.01, and *** = *p* ≤ 0.001). Further, SERPINE1 (-29.6), FOS (-2.25), ANG-2 (-3.18), VEGF (-3.8), and MMP1 (-13.3) were downregulated the most in PC-3 while SERPINE5 (-3.43) and MMP9 (-21.8) were downregulated most in the LNCaP line. In the PC-3M cell line, which is considered an aggressive metastatic mCRPC/NEPC model (Ohnuki et al., 1980), SERPINEB5, ANG-2, VEGF, MMP1, and MMP9 were among the downregulated genes post-METRO-TOPO treatment (Figures 7A–E). Downregulation of proteins representing the top DEGs was also observed in 22Rv1 and DU145 cell lines; however, the fold changes were lower in comparison (Figures 7B–E and Supplementary Table S4).

## Discussion

Previous studies have suggested that a low-dose continuous schedules of chemotherapy holds potential for greater antitumor efficacy and reduced toxicity compared with high doses given intermittently based on the maximum tolerated dose for several cancer types, including prostate (Man et al., 2002; Munoz et al., 2005; Fontana et al., 2009; Nelius et al., 2010). We have demonstrated that metronomic administration of TOPO may be used to achieve increased sensitivity as compared to conventional high-dose drug schedules to overcome drug resistance in metastatic, castration-resistant prostate cancers that show nonendocrine differentiation (Aljuffali et al., 2011). However, the mechanism of action is unknown. Our current study confirmed our previous findings and sought to gain mechanistic insights into antitumor activity following METRO-TOPO. Using a panel of human PCa cell lines, patient cohort data, and TCGA database, we identified baseline (untreated patient cohort and TCGA) and post METRO-treatment (cell lines) gene expression profiles. These signatures were correlated with control (untreated) and CONV treatment signatures. Notably, we also identified METRO gene signatures associated with androgen-responsive and androgen-independence, as well as PCa representing AA *vs* EA patients.

The top DEGs associated with METRO-TOPO treatment included TNF, SERPINB5, CDKN1A, CASP8, FOS, MMP9, RPL13A, ITGA3, B2M, S100A4, and PDGFB. Among these genes, down-regulation of SERPINB5, MMP9, and FOS were associated with improved patient survival.

Baseline SERPINEB5 expression was lower in the PCa patient group with higher survival and higher in the NEPC cell lines. Further, following METRO-TOPO treatment, SERPINEB5 was downregulated in all PCa cell lines. The downregulation was greatest (3.5 to 7.5 folds *vs* 1.8 to 3.5 folds) in aggressive androgen-independent (NEPC) PCa compared to the androgen-sensitive PCa. SERPINEB5 is a regulator of cell proliferation, differentiation, and transformation. Several studies have reported that higher expression of SERPINB5 is associated with poor prognosis and OS in various cancers (Manawapat-Klopfer et al., 2016; Chang et al., 2018). SERPINB5 levels were upregulated in malignant cells by transforming growth factor *β*1 (TGF-*β*1), which induces SERPINB5 expression in cancer cell lines by either a decapentaplegic homolog (Smad)-dependent pathway or by non-Smad signaling pathways via the intermediate signaling molecules MEK1/2 and p38 MAPK(Wongnoppavich et al., 2017). In our study TGF-*β*1 also showed higher expression in the androgen-independent (NEPC) compared to cells that were sensitive to androgen deprivation therapy. These data are interesting and further studies are needed to determine their overall role and importance.

Urokinase-type plasminogen activator (uPA)/urokinase-type plasminogen activator receptor (uPAR) complex expression plays a significant role in invasion and metastasis by increasing production of plasmin from plasminogen leading to ECM degradation. uPA reduces tumor growth by downregulating the MMPs (MMP2 and MMP9) associated with metastasis (Pavón et al., 2016). Serpin family member 1 gene (SERPINE1) is a primary inhibitor of PLAU. PLAU inhibition is involved in regulating cell adhesion and spreading and acts as a regulator of cell migration (Kjøller et al., 1997; Brungs et al., 2017). Several studies have indicated that SERPINE1 expression is associated with poor outcomes, higher grade tumors and increased risk of metastasis in various cancers (Shi et al., 2017; Hsu et al., 2019). On the other hand, higher PLAU and SERPINE1 expression levels significantly reduced Disease Free Survival (DFS) and decreased OS in human cancers (Jevrić et al., 2019). Both SERPINE1 and PLAU levels have also been shown to be elevated in PCa patients (Trabert et al., 2017; Serafin et al., 2018). We observed increased expression of SERPINE1 and PLAU in PC-3 compared to LNCaP (Figure 6; Supplementary Figure S5). SERPINE1 and PLAU are two members of those common gene signature which were elevated in PC-3, PC-3M compared to LNCaP and downregulated differentially after METRO-TOPO treatment (showed by mRNA expression and Immunoblotting). Interestingly, we also found greater expression of SERPINE1 in AA patients compared to EA patients.

Importantly, METRO-TOPO treatment significantly downregulated SERPINE1 (Figure 7) and PLAU (Supplementary Figure S5) expression at the protein level, which may play an important role in increased treatment potency compared to CONV-TOPO treatment schedules. Downregulation of SERPINE1 significantly reduces cellular proliferation by failure to progress from G0/G1 to S phase of the cell cycle (Giacoia et al., 2014). This is consistent with our previous studies where we reported that the treatment of PC-3 cells with METRO-TOPO increased the percentage of cells in G2/M phase with a concomitant decrease in the G1 population and an increase in S phase cell populations (Aljuffali et al., 2011).

FOS is a transcription factor known to promote metastatic PCa and to play an important role in PCa progression and aggressiveness (Rosenberg et al., 2017; Sharma et al., 2018). The MAPK/AP-1 pathway involves upregulation of p-c-fos and p-c-jun as key regulators of cell proliferation in cancer (Zhu et al., 2018). Further, FOS also promotes increased expression of EGFR (HER1/ErbB1) and affects cell differentiation and proliferation during cancer progression (Zhu et al., 2018). Our studies have shown that METRO-TOPO treatment downregulates FOS, MAP2K1 and ERBB2 to a greater extent compared to CONV treatment.

Vascular networks are important for an adequate supply of oxygen and nutrients and the removal of waste products, which is favorable for cell proliferation as well as metastatic spread (Tonini et al., 2003). Various proteins (VEGF, VEGFR, NRP, HIF-*α*, Ang family—Ang1, Ang2, PDGF-BB) and receptors (TGF-b, FGF, HGF, MMPs, PAI-1/SERPINE1) are involved in angiogenesis (Tonini et al., 2003; Domińska et al., 2018; Melegh and Oltean, 2019). An imbalance in vascular endothelial growth factor (VEGF) and angiopoietins (Ang) is the leading cause of disordered structure in tumor vasculature, which promotes cell death, vascular regression, and inflammation (Mazzieri et al., 2011; Fagiani and Christofori, 2013). In contrast, development of immature vessels and neovascularization is associated with poor prognosis (Eklund and Saharinen, 2013; Fagiani and Christofori, 2013). Recent studies have shown that metronomic treatment has anti-angiogenic potential by downregulating pro-angiogenic factors Hif-1α and VEGF resulting in decreased proliferation and micro-vessel density (Merritt et al., 2009; Winter et al., 2016). Our results showed METRO-TOPO downregulated VEGF and ANGPT2 significantly. Further, IPA analysis predicted inhibition of angiogenesis following METRO-TOPO treatment as a top canonical pathway.

Matrix metalloproteinase-1 (MMP1) is involved in cell migration and invasion in PCa (Pulukuri and Rao, 2008; Ozden et al., 2013). MMP9 (extracellular matrix protein) plays a role in invasion, metastasis, promotes the growth of tumor cells in the bone and induces tumor-enhanced bone matrix turnover in PCa (Aalinkeel et al., 2011; Ma et al., 2017). In this study, we demonstrated that MMP1 and MMP9 were downregulated in following METRO-TOPO treatment. Further, enhanced expression of SERPINB5 and MMP9 was associated with low survival in TCGA PRAD (The Cancer Genome Atlas Prostate Adenocarcinoma) patients.

**FIGURE 8:**
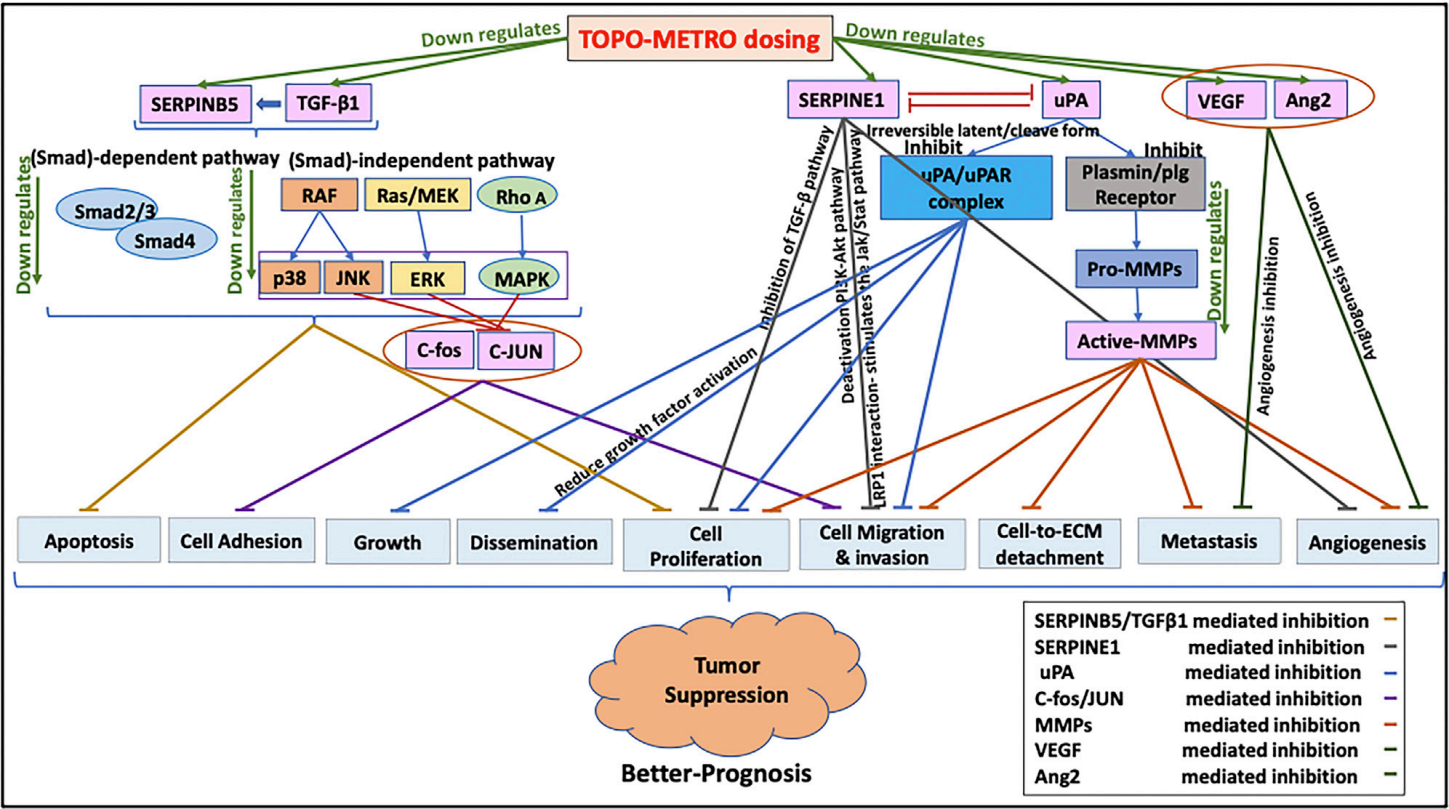
Proposed mechanisms underlying increased potency of METRO-TOPO dosing. METRO-TOPO treatment downregulates the key cancer pathway genes, which are responsible for cell proliferation, migration, invasion, differentiation, transformation, metastasis, and anti-angiogenesis.

Taken together, our results showed METRO-TOPO treatment downregulated 34 key cancer pathway genes responsible for cell proliferation, migration, invasion, differentiation, transformation, metastasis, and anti-angiogenesis indicating a probable basis for the increased potency of METRO-TOPO over CONV dosing (Figure 8). Additionally, the top genes SERPINB5, SERPINE1, CDKN1A, TNF, FOS, ANGPT1, MMP1 and MMP9 were expressed differentially between cell lines, patients representing AA *vs* EA, and significantly associated with patient’s survival.

In conclusion, these data suggest that METRO-TOPO treatment may provide a novel strategy to alter the expression of genes that are associated with tumor growth, metastases, and survival in prostate cancers. An integrated approach that combined *in-vitro* and *ex vivo* discovery followed by *in silico* correlation of molecular signatures associated with METRO response in a diverse panel of PCa, including existing cell lines derived from self-identified AA patients was used. While a variety of PCa cell lines were used, these data represent only a small fraction of genetic and ethnic diversity that is observed clinically. Therefore, larger scale pharmacogenomics studies involving a wider array of patient derived samples are necessary to validate these molecular signatures, as well as *in vivo* animal studies using a range of patient derived organoids or patient derived xenograft models to evaluate METRO-TOPO activity. Overall, these data suggest that METRO-TOPO treatment was effective against aggressive, metastatic, androgen-insensitive NEPC, where there are limited treatment options.

## Data Availability

The original contributions presented in the study are included in the article/Supplementary Material, further inquiries can be directed to the corresponding authors.

## Glossary

22RV1: EA origin, androgen-sensitive human prostate cancer cells
AA: African American or African Americans
C4-2B: EA origin, androgen-sensitive human prostate cancer cells
CONT: Control
CONV: Conventional
CYC: Cyclophosphamide
DEG: Differentially expressed gene
DFS: Disease-free survival
DMSO: dimethyl sulfoxide
DU145: EA origin, metastatic, castration-resistant human prostate cancer cells that show neuroendocrine differentiation (NEPC)
DUTXR: EA origin DU145 taxane resistant human prostate cancer cells
EA: European American or Caucasian
FBS: Fetal bovine serum
FDR: False discovery rate
FPKM: Fragments Per Kilobase of transcript per Million mapped reads
GEP: Gene expression profiling
IC_50_: The half-maximal inhibitory drug concentration
IPA: Ingenuity Pathway Analysis
LNCaP: EA origin, androgen-dependent human prostate cancer epithelial cells
mCRPC: Metastatic, castration-resistant prostate cancer: Metastatic, castration-resistant prostate cancer
MDA-Pca-2b: AA origin, androgen-independent human prostate cancer epithelial cells
METRO: Metronomic
mRNA: Messenger RNA
MTT: 3-(4, 5-Dimethylthiazol-2-yl)-2,5-diphenyltetrazolium bromide
mCRPC: Metastatic, castration-resistant prostate cancer: Metastatic, castration-resistant prostate cancer
NEPC: Neuroendocrine Prostate Cancer
nmCRPC: Non-metastatic, castration-resistant prostate cancer
OS: Overall survival
PBS: Phosphate buffered saline
PC-3: EA origin, metastatic, castration-resistant human prostate cancer cells that show neuroendocrine differentiation (NEPC)
PC-3M: EA origin, highly metastatic, castration-resistant human prostate cancer cells that show neuroendocrine differentiation (NEPC)
PCa: Prostate cancer
RNA-Seq: RNA sequencing
SRB: Sulforhodamine
BTBS: Tris-buffered saline
TCA: Trichloroacetic acid
TCGA: The Cancer Genome Atlas
TOPO: Topotecan
TRIS: Tris (hydroxymethyl) aminomethane buffer
