# Supplementary material for: Identification and Characterization of Key Differentially Expressed Genes Associated With Metronomic Dosing of Topotecan in Human Prostate Cancer
Source: Front Pharmacol. 2021 Dec 6;12:736951. doi: 10.3389/fphar.2021.736951 (PMC8685420; doi:10.3389/fphar.2021.736951)

**Supplementary Figure 4. A, B, C, D, E**

Immunoblotting of top significant genes for METRO-TOPO treatment in LNCaP, PC-3, PC-3M, 22RV1 and DU145; BETA ACTIN was used as a housekeeping gene (positive control). Consistently higher downregulation of candidate genes was observed for METRO treatment in LNCaP and PC-3.

**A, B)** Densitometry plots for top significant proteins for METRO-TOPO dosing (SERPINB5, SERPINE1, FOS, ANG-2, VEGF, MMP-1, MMP9) were differentially expressed significantly in CONV vs METRO in LNCaP and PC-3 cell lines (p * = p ≤ 0.05, ** = p ≤ 0.01, *** = p ≤ 0.001). Protein expression of these top significant proteins was higher in PC-3 (mCRPC) compared to LNCaP (less aggressive) cells. Additionally, METRO-TOPO treatment downregulated all these top proteins to a greater extent in PC-3 compared to LNCaP cells.

**C, D, E)** Significant genes (SRPINEB5, ANG-2, VEGF, MMP-1, and MMP-9) were downregulated for METRO treatment in PC-3M cell lines which consider was a better mCRPC (PubMed;7471073). model. Downregulation of top significant genes in 22RV1 and DU145 was also observed, however downregulation was less compared to other cell lines (**Table S2).**

**A)**

**
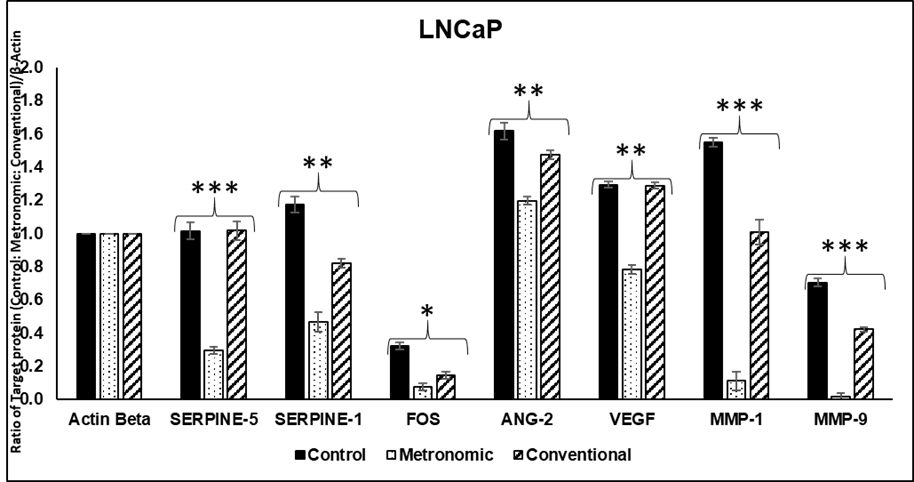
**

**B)**

**
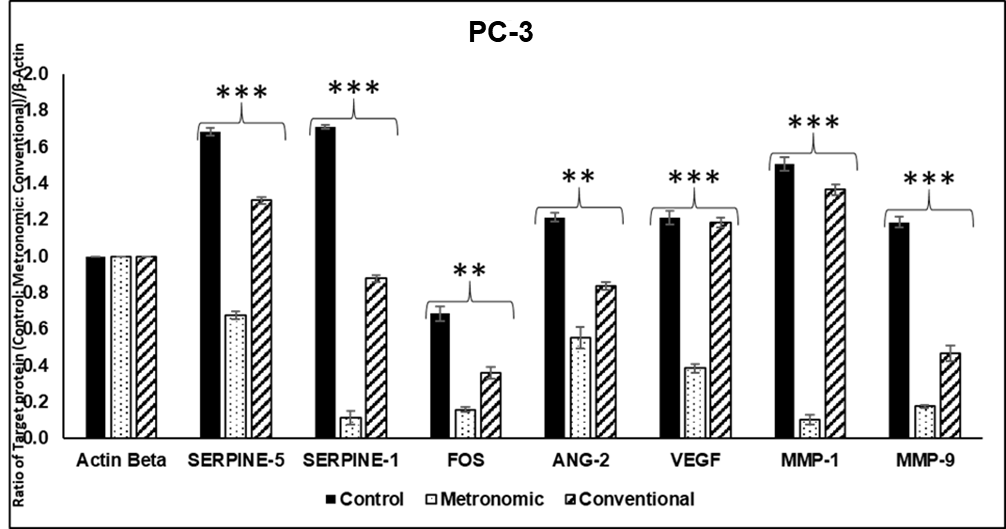
**

**C)**

**
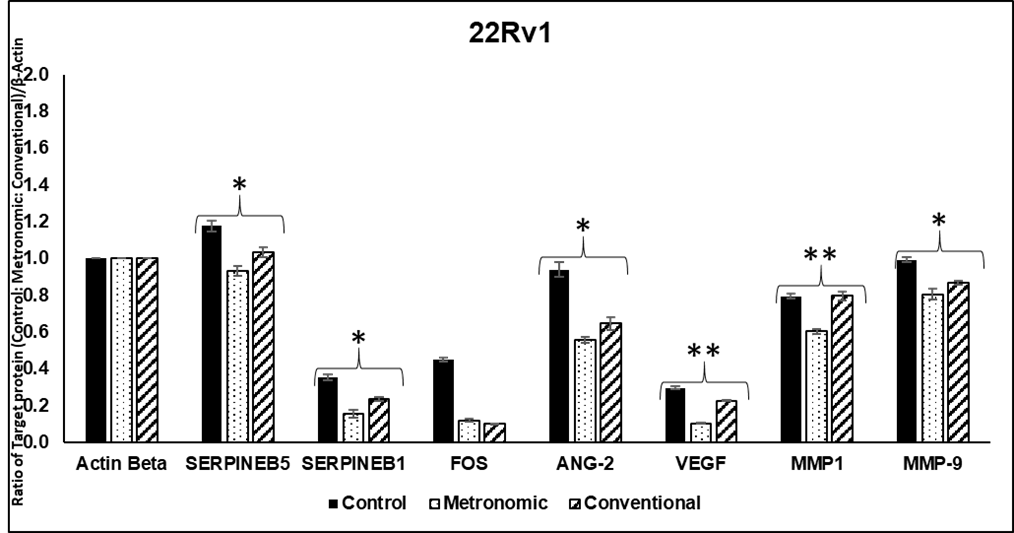
**

**D)**

**
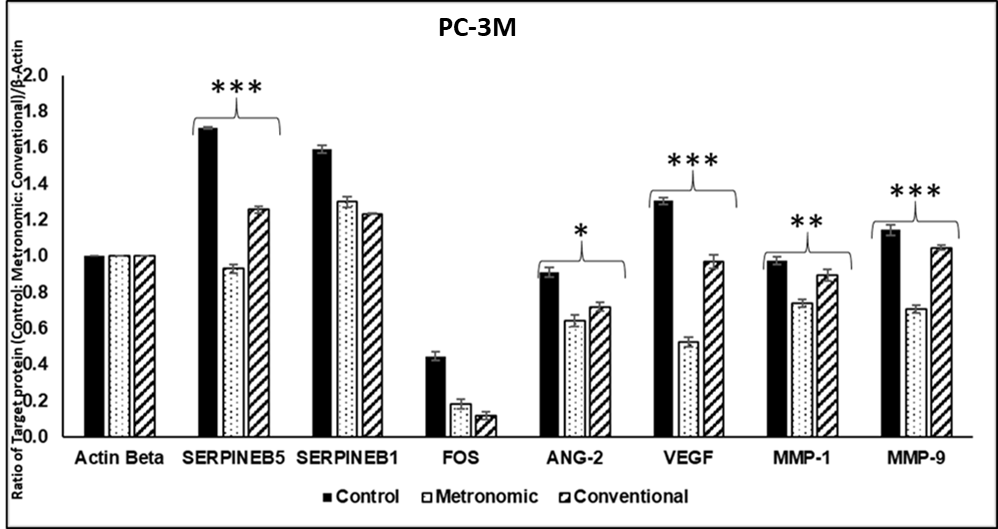
**

**E)**


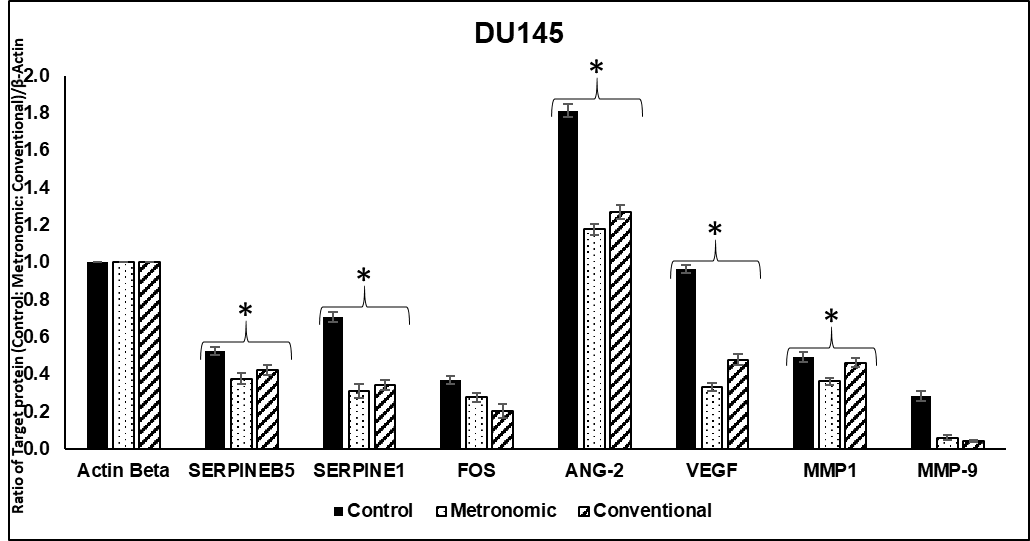

Supplement: Supplementary file 1 [file DataSheet4.docx]
