# Supplementary material for: Identification and Characterization of Key Differentially Expressed Genes Associated With Metronomic Dosing of Topotecan in Human Prostate Cancer
Source: Front Pharmacol. 2021 Dec 6;12:736951. doi: 10.3389/fphar.2021.736951 (PMC8685420; doi:10.3389/fphar.2021.736951)
Supplement: Supplementary file 2 [file Table1.docx]

| **Gene** | **Gene expressed at 48 h CONV-TOPO treatment for PC-3 cell line** | **Gene expressed at 48 h METRO-TOPO treatment compare to TOPO-CONV treatment for PC-3 cells** | **Gene expressed at 48 h CONV-TOPO treatment for LNCaP cell line** | **Gene expressed at 48 h METRO-TOPO treatment compare to CONV-TOPO treatment for LNCaP cells** |
| --- | --- | --- | --- | --- |
| CDKN1A | 11.5 | -8.3 | 13.4 | -8.3 |
| FGFR2 | 6.5 | -4.3 | 2.9 | -2.1 |
| SERPINB5 | 3.7 | -3.9 | 13.2 | -8.4 |
| FAS | 3.1 | -3.7 | 5.6 | -5.5 |
| ITGB3 | 14 | -9.6 | NA | NA |
| CXCL8 | 7.3 | -3.3 | NA | NA |
| MMP2 | 6.5 | -4.3 | NA | NA |
| TERT | 6.5 | -4.3 | NA | NA |
| TIMP3 | 6.5 | -4.3 | 2.5 | NA |
| FOS | 5.2 | -2.8 | NA | -3.8 |
| IFNB1 | 5.1 | -2.8 | NA | NA |
| GZMA | 5 | -2.5 | NA | NA |
| MTSS1 | 4.6 | -3.6 | NA | NA |
| IGF1 | 4.3 | -2.6 | -2 | NA |
| SNCG | 3.8 | -4.1 | NA | NA |
| THBS1 | 3.8 | -2.4 | 2.1 | NA |
| MMP1 | 3.1 | -3 | NA | NA |
| ITGA4 | 2.6 | -2.7 | NA | NA |
| CDC25A | 2.6 | NA | NA | NA |
| CDKN2A | 2.5 | -2.9 | NA | NA |
| B2M | 2.4 | -2.9 | NA | NA |
| CDK2 | 2.4 | -2.6 | NA | NA |
| MAP2K1 | 2.4 | -2.3 | NA | NA |
| EPDR1 | 2.3 | -2.1 | NA | NA |
| PDGFA | 2.2 | NA | 2.1 | NA |
| CASP8 | 2.2 | -2.1 | NA | -2.8 |
| ITGAV | 2.2 | -2.2 | NA | NA |
| ETS2 | 2.1 | -2 | NA | NA |
| TIMP1 | 2 | -2 | NA | NA |
| NFKB1 | 2 | -2.2 | NA | NA |
| CFLAR | 2 | NA | NA | NA |
| MMP9 | 2 | NA | NA | -2.2 |
| BCL2L1 | -2 | NA | NA | NA |
| SERPINE1 | -2.1 | NA | NA | -3.3 |
| MYC | -2.1 | 2.5 | NA | NA |
| IFNA1 | -2.7 | 2.5 | NA | NA |
| TNF | NA | -2.6 | 4.1 | -5.8 |
| MDM2 | NA | NA | 3.4 | -3.3 |
| ANGPT2 | NA | NA | 3.4 | -3.2 |
| ITGA3 | NA | NA | 3.3 | -3.3 |
| JUN | NA | NA | 3.2 | -2.5 |
| BAX | NA | NA | 2.6 | -2.9 |
| RPL13A | NA | NA | 2 | -2.1 |
| E2F1 | NA | NA | -2.5 | NA |
| PLAUR | NA | NA | -3.2 | NA |
| TEK | NA | NA | NA | NA |
| **ANGPT1** | 2.8 | 2.8 | NA | NA |
| **HPRT1** | NA | -2 | NA | NA |

**Supplementary Table 1**. Top differentially expressed genes (DEGs) following 72 h CONV and METRO-TOPO treatment for PC-3 and LNCaP cell lines. Expression level of genes were assessed by qRT-PCR (RT² Profiler™ PCR Array Human Cancer PathwayFinder (PAHS-033A by QIAGEN). Gene expression level of treatment group compared with no drug treatment group and also between CONV vs METRO treatment group for both the cell lines at 48 h. Gene expression in the treatment group were normalized to corresponding control groups (no drug treatment for both PC-3 and LNCaP cell lines). Fold change cut-off value was >2. CDKN1A, FGER2, FAS and SERPINB5 genes were expressed in both treatment groups but there was variation in induction/suppression of expression level - downregulated in the METRO treatment group, whereas they were up-regulated in the CONV treatment group.

NA-Fold regulation is < 2.
