# Supplementary material for: Identification and Characterization of Key Differentially Expressed Genes Associated With Metronomic Dosing of Topotecan in Human Prostate Cancer
Source: Front Pharmacol. 2021 Dec 6;12:736951. doi: 10.3389/fphar.2021.736951 (PMC8685420; doi:10.3389/fphar.2021.736951)
Supplement: Supplementary file 3 [file DataSheet3.docx]

**Supplementary Figure 3. A, B, C, D. Ingenuity pathway analysis predictions (Table S3)**

A) Prostate cancer signaling, PTEN, IL-8 and P53 signaling as a target for TOPO METRO-72 h treatment in PC-3 cell line (representing mCRCP).

B) IL-8 signaling and P53 signaling pathway as a target for METRO-TOPO -72 h treatment in LNCaP cell line.

C) Tumor inhibition pathway as target for METRO-TOPO vs CONV treatment in LNCaP and PC-3 cell line.

D) Predicted top upstream network, where MMP1 was identified as a key regulatory factor for the cancer pathway for LNCaP and PC-3 cell lines for METRO-TOPO treatment.


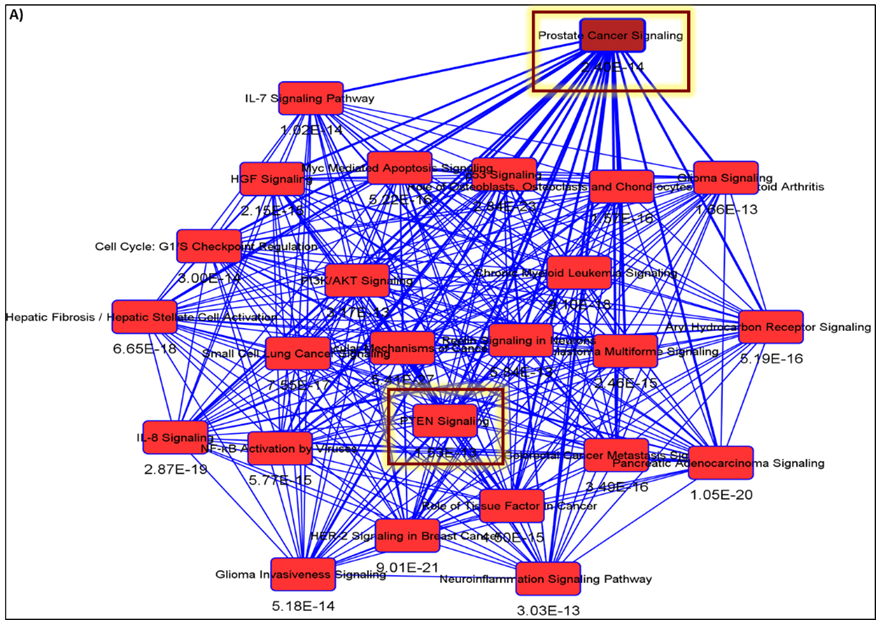


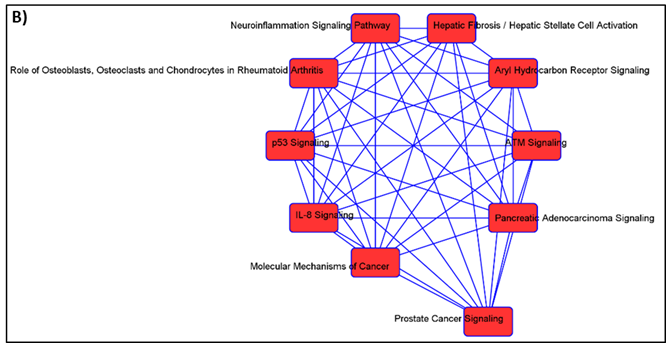


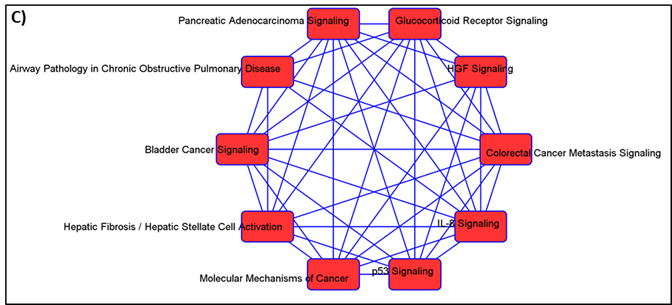


**D)**

**
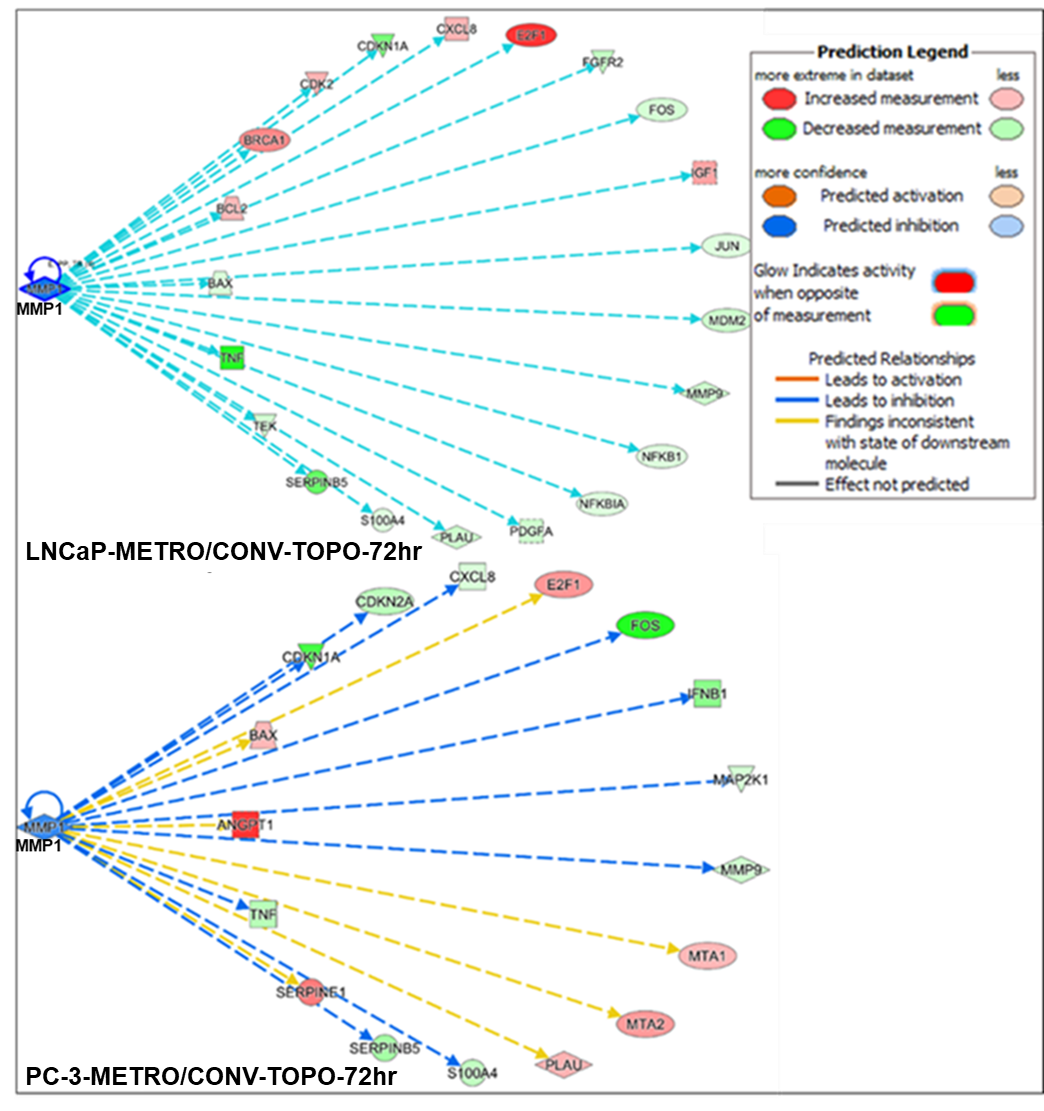
**
