# Supplementary material for: Identification and Characterization of Key Differentially Expressed Genes Associated With Metronomic Dosing of Topotecan in Human Prostate Cancer
Source: Front Pharmacol. 2021 Dec 6;12:736951. doi: 10.3389/fphar.2021.736951 (PMC8685420; doi:10.3389/fphar.2021.736951)
Supplement: Supplementary file 4 [file DataSheet5.docx]

**Supplementary Figure 5.** Immunoblotting of the top significant PLAU gene following TOPO-METRO treatment in LNCaP, PC-3; BETA ACTIN was used as a housekeeping gene (positive control). Consistently higher downregulation of this candidate gene was observed for METR treatment in LNCaP and PC-3.

**
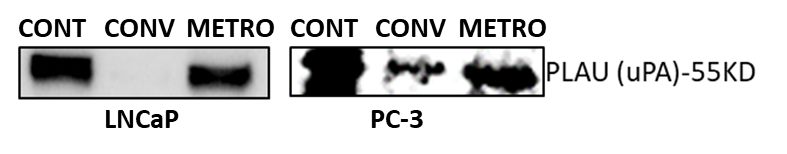
**
