# Supplementary material for: Identification and Characterization of Key Differentially Expressed Genes Associated With Metronomic Dosing of Topotecan in Human Prostate Cancer
Source: Front Pharmacol. 2021 Dec 6;12:736951. doi: 10.3389/fphar.2021.736951 (PMC8685420; doi:10.3389/fphar.2021.736951)
Supplement: Supplementary file 5 [file Table2.docx]

| **Top canonical pathway** | **P-Value** |
| --- | --- |
| IL-7 Signaling Pathway | 1.02E-14 |
| HGF Signaling Pathway | 1.15E-15 |
| Myc Mediated Apoptosis signaling | 5.22E-16 |
| P53 signaling Pathway | 2.34E-23 |
| Cell Cycle: G1/S Checkpoint Regulation | 3.00E-14 |
| PI3K/AKT Signaling | 3.17E-13 |
| Ary Hydrocarbon Receptor Signaling | 5.19E-20 |
| PTEN signaling | 1.53E-13 |
| IL-8 Signaling | 2.87E-19 |
| NFκβ- Activation | 5.77E-15 |
| HER-2 Signaling in Breast cancer | 4.05E-15 |
| Neuroinflamation Signaling | 3.03E-13 |
|  |  |
| **Top diseases** | **P-Value** |
| Prostate Cancer Signaling | 1.40E-14 |
| Glioma Signaling | 1.66E-13 |
| Chronic Leukemia Signaling | 9.10E-18 |
| Hepatic Fibrosis | 6.65E-18 |
| Small Cell Lung Cancer* | 7.55E-17 |
| Colorectal Cancer | 3.49E-16 |
| Pancreatic adenocarcinoma | 1.05E-20 |

**Supplementary Table 2.** Ingenuity pathway analysis (IPA) results based on top DEGs for METRO-TOPO treatment in PCa cell lines.

* TOPO is a FDA approved drug for Small Cell Lung Cancer
