# Supplementary material for: Identification and Characterization of Key Differentially Expressed Genes Associated With Metronomic Dosing of Topotecan in Human Prostate Cancer
Source: Front Pharmacol. 2021 Dec 6;12:736951. doi: 10.3389/fphar.2021.736951 (PMC8685420; doi:10.3389/fphar.2021.736951)
Supplement: Supplementary file 6 [file Table3.docx]

| **Gene** | **Fold-Change (HIGH *vs.* LOW survival)** |
| --- | --- |
| FOS | 2.20E+34 |
| SERPINB5 | -2.99426 |
| CCNE1 | -1.52035 |
| TWIST1 | 268.504 |
| MMP9 | 3.25532 |
| MCAM | -24.238 |
|  |  |
| **Gene** | **Fold-Change (AA *vs*. EA)** |
| MMP1 | 2.19933 |
| B2M | 2.48E+50 |
| CXCL8 | 18.0908 |
| PDGFA | -3.41545 |
| ERBB2 | 15.5559 |
| ITGA1 | -3.08789 |
| ITGA3 | -2.74333 |
| JUN | -1.19E+11 |

**Table S3.** *In silico* validation of top METRO-TOPO treatment-associated DEGs using prostate cancer patient data from the TCGA database.

HIGH *vs* LOW survival; AA (African American) *vs* EA (European American) men.
