# Supplementary material for: Identification and Characterization of Key Differentially Expressed Genes Associated With Metronomic Dosing of Topotecan in Human Prostate Cancer
Source: Front Pharmacol. 2021 Dec 6;12:736951. doi: 10.3389/fphar.2021.736951 (PMC8685420; doi:10.3389/fphar.2021.736951)

**Supplementary Figure 2.** **A, B, C, D.**

Dot plots representing the fold change between cell lines; genes expressed in Castration-resistant/NEPC cell line PC-3 and castration-sensitive cell line LNCaP normalized to the control (No -drug treatment for each time point; control 48 and 72 h respectively). Fold change cut-off value >2 (Log2 ratio). Pink represents gene expression for metronomic *vs* blue representing gene expression for conventional treatment.

mRNA expression for conventional and metronomic A) 48 h treatment B) 72 h treatment in PC-3 the cell line treated with topotecan; C) 48 h treatment and D) 72 h treatment in the LNCaP cell line treated with topotecan.


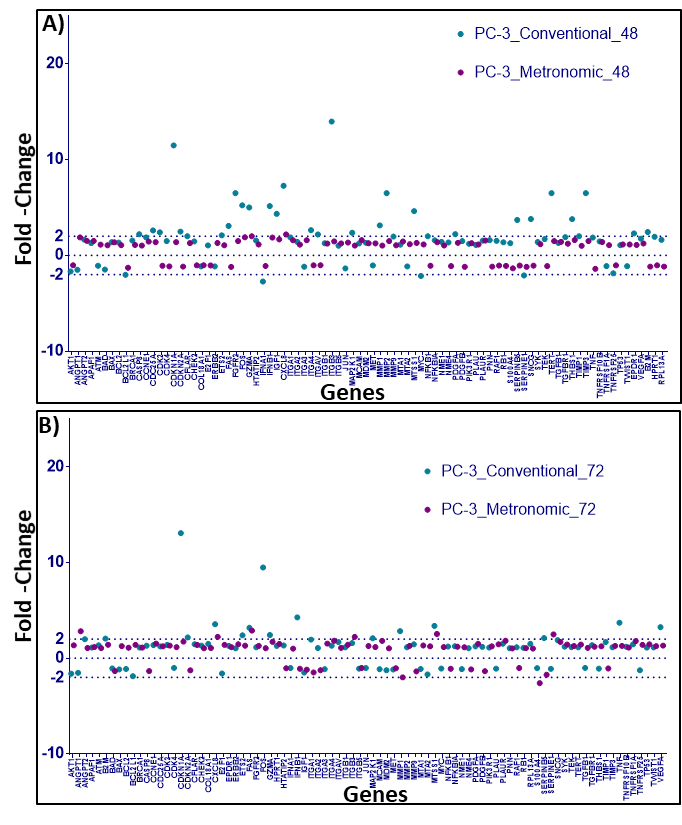


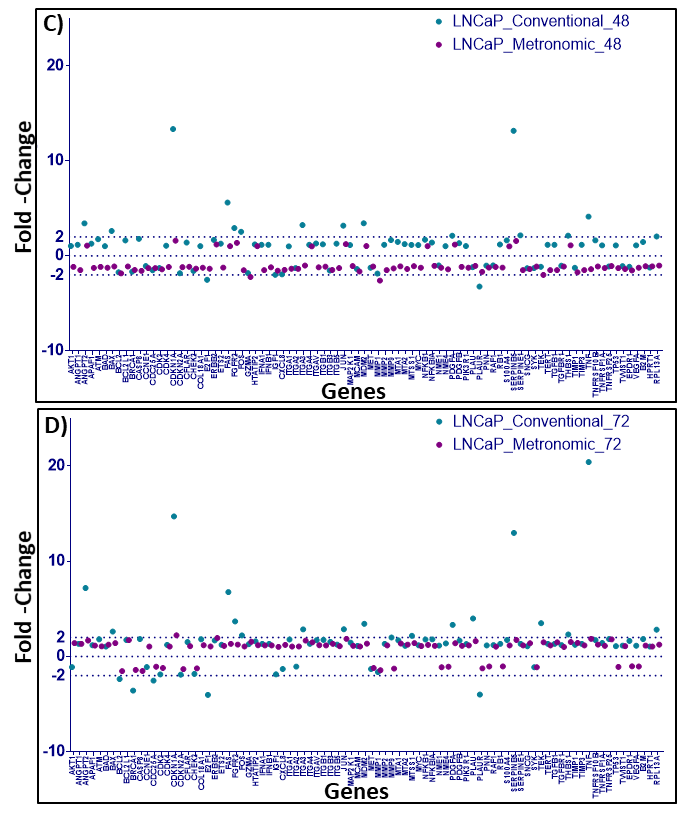

Supplement: Supplementary file 7 [file DataSheet2.docx]
