# Supplementary material for: Identification and Characterization of Key Differentially Expressed Genes Associated With Metronomic Dosing of Topotecan in Human Prostate Cancer
Source: Front Pharmacol. 2021 Dec 6;12:736951. doi: 10.3389/fphar.2021.736951 (PMC8685420; doi:10.3389/fphar.2021.736951)
Supplement: Supplementary file 8 [file Table4.docx]

| **Protein** | **Cell line** | **CONT** | **METRO** | **CONV** | **METRO/CONV (Fold-Change)** |
| --- | --- | --- | --- | --- | --- |
| **SERPINEB5** | LNCaP | 1.02 | 0.30 | 1.02 | 3.43* |
|  | PC3 | 1.68 | 0.68 | 1.31 | 3.20 |
|  | PC3M | 1.71 | 0.93 | 1.26 | 2.50 |
|  | 22Rv1 | 1.18 | 0.93 | 1.03 | 1.43 |
|  | DU145 | 0.53 | 0.38 | 0.42 | 1.74 |
| **SERPINE1** | LNCaP | 1.18 | 0.47 | 0.82 | 3.61 |
|  | PC3 | 1.71 | 0.11 | 0.88 | 29.59* |
|  | PC3M | 1.59 | 1.30 | 1.23 | 1.58 |
|  | 22Rv1 | 0.35 | 0.15 | 0.24 | 3.36 |
|  | DU145 | 0.71 | 0.31 | 0.34 | 4.69 |
| **FOS** | LNCaP | 0.32 | 0.08 | 0.14 | 1.92 |
|  | PC3 | 0.69 | 0.16 | 0.36 | 2.25* |
|  | PC3M | 0.45 | 0.18 | 0.12 | 0.65 |
|  | 22Rv1 | 0.45 | 0.12 | 0.10 | 0.85 |
|  | DU145 | 0.37 | 0.28 | 0.20 | 0.73 |
| **ANG-2** | LNCaP | 1.62 | 1.20 | 1.47 | 1.48 |
|  | PC3 | 1.21 | 0.55 | 0.84 | 3.18* |
|  | PC3M | 0.91 | 0.64 | 0.72 | 1.80 |
|  | 22Rv1 | 0.94 | 0.56 | 0.65 | 2.44 |
|  | DU145 | 1.81 | 1.18 | 1.27 | 2.20 |
| **VEGF** | LNCaP | 1.30 | 0.78 | 1.29 | 1.65 |
|  | PC3 | 1.21 | 0.39 | 1.19 | 3.08* |
|  | PC3M | 1.30 | 0.53 | 0.97 | 1.84 |
|  | 22Rv1 | 0.30 | 0.10 | 0.23 | 2.18 |
|  | DU145 | 0.96 | 0.33 | 0.48 | 1.44 |
| **MMP1** | LNCaP | 1.55 | 0.11 | 1.01 | 8.93 |
|  | PC3 | 1.51 | 0.10 | 1.37 | 13.25* |
|  | PC3M | 0.98 | 0.74 | 0.89 | 1.21 |
|  | 22Rv1 | 0.79 | 0.60 | 0.80 | 1.32 |
|  | DU145 | 0.49 | 0.36 | 0.46 | 1.27 |
| **MMP9** | LNCaP | 0.71 | 0.02 | 0.42 | 21.84* |
|  | PC3 | 1.19 | 0.18 | 0.47 | 2.64 |
|  | PC3M | 1.14 | 0.71 | 1.05 | 1.49 |
|  | 22Rv1 | 0.99 | 0.80 | 0.87 | 1.08 |
|  | DU145 | 0.29 | 0.06 | 0.04 | 0.71 |

**Table S4.** Western blotting: Protein expression for Control (CONT), conventional (CONV) and metronomic (METRO) treatment at 72 h for LNCaP, PC-3, PC-3M, 22RV1 and DU145 cell lines treated by TOPO. Expression levels of proteins were assessed (Immunoblotting) by gene-specific antibody binding. Beta actin was used as a loading control gene. Expression level of treatment group were compared with no drug treatment group and also between CONV vs METRO treatment group for all cell lines at 72 h. SERPINE1 (29.59), FOS (2.25), ANG-2 (3.18), VEGF (3.8) and MMP1 (13.25) were downregulated to the greatest extent in PC-3 cells for METRO-TOPO treatment whereas SERPINE5 (3.43) and MMP9 (21.84) were downregulated most in LNCaP cells.
