# Supplementary material for: Identification and Characterization of Key Differentially Expressed Genes Associated With Metronomic Dosing of Topotecan in Human Prostate Cancer
Source: Front Pharmacol. 2021 Dec 6;12:736951. doi: 10.3389/fphar.2021.736951 (PMC8685420; doi:10.3389/fphar.2021.736951)

**Supplementary Figure 1. Effect of topotecan on the growth of prostate cells *in vitro* following Conventional and Metronomic dosing.**

A) *In vitro* cytotoxicity was assessed following 48 and 72 h of CONV or METRO treatment in PC-3M, DU145, DUTXR and 22Rv1 cell lines using concentration-dependent decrease in protein staining (SRB) at increasing drug concentrations.

B) Microscope images showing treatment effect on the cell lines PC-3 and LNCaP. Results show significantly higher cell death in METRO compared to CONV treatment for both the cell lines; ImageJ analysis showed significant difference in cell density for CONV vs METRO treatment in PC-3 and LNCaP cell lines. (Significant P value * = p ≤ 0.05).

**A**

**
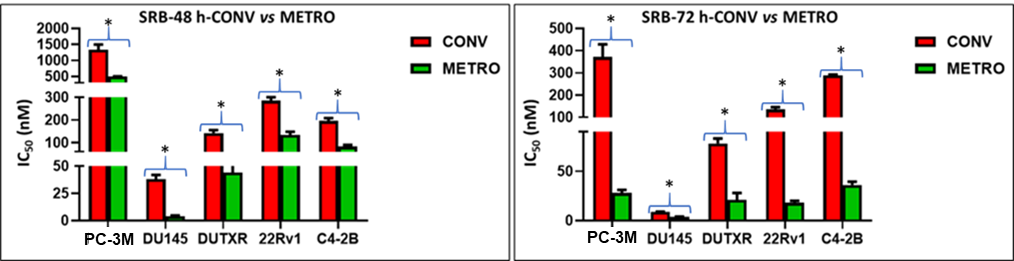
**

**B**


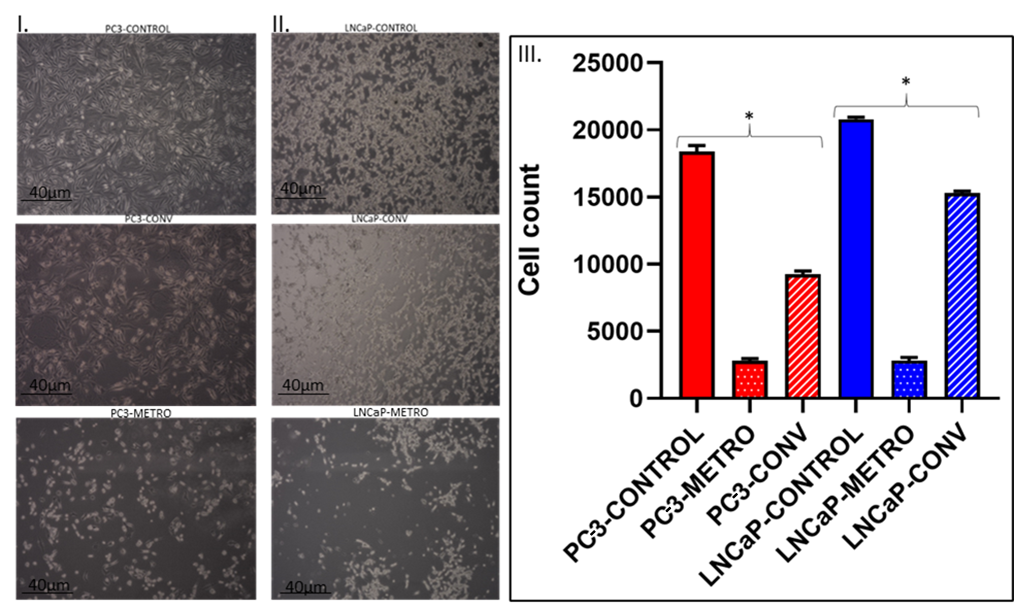

Supplement: Supplementary file 9 [file DataSheet1.docx]
